# Allocation of Space-Based Attention is Guided by Efficient Comprehension of Spatial Direction

**DOI:** 10.5334/joc.325

**Published:** 2024-01-08

**Authors:** Adam J. Barnas, Natalie C. Ebner, Steven M. Weisberg

**Affiliations:** 1 Department of Psychology, University of Florida, Gainesville, FL, USA

**Keywords:** space-based attention, central cue, cue validity effect, spatial cognition, spatial navigation

## Abstract

Spatial navigation is supported by visual cues (e.g., scenes, schemas like arrows, and words) that must be comprehended quickly to facilitate effective transit. People comprehend spatial directions faster from schemas and words than scenes. We hypothesize that this occurs because schemas and words efficiently engage space-based attention, allowing for less costly computations. Here, participants completed a spatial cueing paradigm, and we calculated cue validity effects – how much faster participants responded to validly than invalidly cued locations – for each cue format. We pre-registered Experiment 1 and found significant cue validity effects with schemas and words, but not scenes, suggesting space-based attention was allocated more efficiently with schemas and words than scenes. In Experiment 2, we explicitly instructed participants to interpret the scenes from an egocentric perspective and found that this instruction manipulation still did not result in a significant cue validity effect with scenes. In Experiment 3, we investigated whether the differential effects between conditions were due to costly computations to extract spatial direction and found that increasing cue duration had no influence. In Experiment 4, significant cue validity effects were observed for orthogonal but not non-orthogonal spatial directions, suggesting space-based attention was allocated more efficiently when the spatial direction precisely matched the target location. These findings confirm our hypothesis that efficient allocation of space-based attention is guided by faster spatial direction comprehension. Altogether, this work suggests that schemas and words may be more effective supports than scenes for navigation performance in the real-world.

## Introduction

Spatial directions, conveyed through formats like schemas (i.e., arrows), words (i.e., “left” or “right”) or visual scenes (i.e., maps), help people determine which way to go. Efficiently extracting spatial directions from a visually cluttered environment can make navigation safer and less error prone, for example when driving down a busy highway with many on- and off-ramps or searching for the baggage claim in a crowded airport terminal. Spatial direction is comprehended more quickly with some formats than others, but the cognitive mechanism underlying this facilitation has not been identified yet. Here, we propose a role for spatial direction comprehension in the allocation of space-based attention and test the hypothesis that allocation of space-based attention is guided by efficient comprehension of spatial direction for cues that match a prepotent representational format, like arrows and words, compared to other formats, like maps or scenes.

Attentional mechanisms serve to highlight relevant information and filter out distractions. Building on the spatial receptive field organization of the visual system (Hubel & Wiesel, 1959), the focus of external visual attention is believed to be primarily space-based. That is, the information to which one attends is selected based upon its location in the visual field. As a result, directing attention to a specific spatial location allows an individual to more deeply and efficiently process (as measured by speeded responses or heightened accuracy) visual information at an attended location versus an unattended location (Eriksen & Eriksen, 1974; Posner, 1980; Posner, Snyder, & Davidson, 1980; for a review, see Carrasco, 2011).

Research on space-based attention often uses the Posner spatial cueing task (Posner, 1980). In this task, participants fixate on a central cross that is flanked on the left and right by outlined square placeholders, each indicating a spatial location where the target could appear. Participants are then cued to one of the two placeholders (left or right). Cues vary widely across studies. C*entral cues* include arrows (for a review, see Chica, Martín-Arévalo, Botta, & Lupiáñez, 2014), eye gaze (for reviews, see Birmingham & Kingstone, 2009; Frischen, Bayliss, & Tipper, 2007), and verbal directions, such as “left”, “right”, “top”/“above”, and “bottom”/“below” (e.g., Hommel et al., 2001). *Peripheral cues* occur at the placeholders and include the abrupt appearance of a small object or the brightening/thickening of target placeholders (e.g., Folk, Remington, & Johnston, 1992). The cue can be *spatially predictive* of a target’s location, meaning that the target appears in the location indicated by the cue on a majority of trials (70–80%) and in the opposite location indicated by the cue on a minority of trials (20–30%); or the cue can be *spatially non-predictive* of a target’s location, meaning that the target appears equally often in the location indicated by the cue and in the opposite location as indicated by the cue (i.e., 50% with two placeholders, 25% with four placeholders, etc.). The target can appear in the placeholder on the left, in the placeholder on the right, or not at all.

There are three main trial types defined by the direction or location indicated by the cue and the location of the target – valid, invalid, and catch. On *valid trials*, the location indicated by the cue and the location of the target match (e.g., a target appears on the right side following a central arrow cue that points to the right). On *invalid trials*, the location indicated by the cue and the location of the target do not match (e.g., a target appears on the right side following an abrupt onset cue that appears on the left). On *catch trials*, which encourage selective responding by the participant and help ensure proper task completion, no target appears following the cue. Additionally, there are *neutral trials*, defined by a cue that highlights a location where the target is never presented or all locations where the target could appear. As a result, neither the left nor right target placeholder benefits from a preferential allocation of space-based attention. On a neutral trial, the target has an equal probability of appearing in the left or right placeholder, or not at all (i.e., a catch trial).

For spatially predictive cues, which bias participants’ space-based attention to one location over others, response time (RT) is typically faster and accuracy higher for targets on valid (i.e., targets appearing at the cued location) than invalid (i.e., targets appearing at the non-cued location) trials, a phenomenon referred to as a *cue validity effect*. This speeding up of RT and enhancement of accuracy are the result of a benefit afforded to validly cued locations due to the preferential allocation of space-based attention. In other words, cues engage attentional mechanisms and direct participants’ space-based attention to the location indicated by the cue. As such, when a target appears in the cued location (valid trial), participants are faster and more accurate to respond to the target because their attention has already been allocated to the location indicated by the cue. Conversely, when a target appears in the non-cued location (invalid trial), participants must re-allocate their space-based attention because the target appears in a different location than where their attention was initially directed, resulting in slower and less accurate responses on invalid trials. Additionally, since the target is equally likely to appear on the left or right side with a neutral cue, RT and accuracy on neutral trials usually fall in between valid and invalid trials (Posner, 1980; Posner et al., 1980).

Space-based attention relies on pre-existing associations between visual information and spatial information. An arrow pointing to the left or the word “left” is more automatically associated with a target on the left even if the target is equally likely to appear in other locations (Hommel, Pratt, Colzato, & Godijn, 2001). Hommel and colleagues presented participants with arrows and words indicating one of four possible spatial locations (up, down, left, right) in a variant of the Posner spatial cueing task. Participants detected targets significantly faster when they appeared in the location indicated by the cue (i.e., a significant cue validity effect) even when the cue was spatially non-predictive of the location of the target.

But does the format of the central cue modulate the allocation of space-based attention? Work from our lab, for example, has demonstrated a performance difference between arrows and words. Weisberg, Marchette, and Chatterjee (2018) presented participants with seven unique spatial directions (ahead, left, right, sharp left, sharp right, slight left, and slight right) in the form of schemas (i.e., arrows), words, and scenes (Google Maps images of roads) in a rolling one-back task. On every trial, participants had to indicate whether the spatial direction was the same or different as the one from the previous trial, regardless of whether the format of the spatial directions differed on consecutive trials. RTs were significantly faster for schemas than for words and scenes, and significantly faster for words than scenes (Weisberg, Marchette, & Chatterjee, 2018; see also Weisberg & Chatterjee, 2021). This pattern of results was observed when RTs were collapsed across the format of the previous trial as well as when the format matched on consecutive trials. Relative to words, spatial direction was comprehended the fastest with schemas and the slowest with scenes.

In an attention context, Gibson and Kingstone (2006) presented participants with four different types of cues (arrows, words, eye-gazes, and peripheral abrupt onsets) in another variant of the Posner spatial cueing task. The authors found an effect of cue type characterized by significantly slower RTs with word cues than arrow, eye-gaze, and abrupt onset cues. However, cues were 100% valid in this study, meaning that the intended spatial direction of the cue and target location matched on every trial, so the efficiency with which distinct cue formats modulated space-based attention, measured as the cue validity effect, could not be computed.

Overall, these findings suggest a model of attentional cueing in which cues that have a stronger pre-existing association with the location of the target may yield an attentional advantage. To our knowledge, scenes, like the ones developed by Weisberg and colleagues (2018) have not been used as cues in the Posner spatial cueing paradigm. It is unknown if and how scenes (and maps) engage visual attention, considering that maps of route perspective (first-person views) and survey perspective (overhead views) displays are widely used for effective transit in the real world (Dai, Thomas, & Taylor, 2018) and that spatial navigation depends on other cognitive (i.e., attention and memory) and perceptual processes (Epstein, Patai, Julian, & Spiers, 2017). Despite this knowledge gap, previous work has shown that spatial navigation is more efficient with external aids like maps and verbal directions than without these aids (Ishikawa, Fujiwara, Imai, & Okabe, 2008; Krukar, Anacta, & Schwering, 2020), and that maps depicting overhead views of an environment were equally effective in enhancing spatial knowledge as route-based verbal directions (Jaeger, Weisberg, Nazareth, & Newcombe, 2023). However, in line with the findings from Weisberg and colleagues (2018), we hypothesize that, due to their visual complexity, scenes as cues for spatial directions may have weaker pre-existing associations with target locations than more simple schemas or to a lesser extent words.

Here, we report a series of experiments designed to investigate the hypothesis that allocation of space-based attention is guided by efficient comprehension of spatial direction, and to determine the extent to which this effect is modulated by spatial direction format. We pre-registered three predictions: (1) faster performance on valid than invalid trials; (2) faster overall performance with schema cues than word and scene cues; and (3) modulation of cue validity effects by cue format. To preview our main result, space-based attention was significantly modulated (measured as a cue validity effect) with schema and word cues, but not scene cues, which supports our hypothesis that schemas and words efficiently engage space-based attention, constituting a cognitive mechanism by which rapid comprehension of spatial direction underlies the allocation of space-based attention. Thus, overlearned communicative symbols, like arrows, and verbal directions may be more effective supports than map-like representations of turns for successful spatial navigation performance in real-world settings.

## Experiment 1 – Cue Format

The goal of Experiment 1 was to investigate whether the format of the central cue (scenes, schemas, and words conveying left, right, or ahead directions) used in Posner’s spatial cueing paradigm (Posner, 1980; Posner et al., 1980) modulated space-based attention. We pre-registered the prediction that the cue validity effect would be the largest with schema cues and smallest with scene cues. Evidence to support this prediction would suggest that preferential allocation of space-based attention to information in relevant spatial locations is associated with the speed by which spatial direction is extracted from navigation cues (see Weisberg et al., 2018).

### Method

#### Participants

We conducted *a priori* power analyses/simulations using the *paramtest* package (Hughes, 2022) in R for each hypothesis.^1^ (For details, see the pre-registration materials on Open Science Framework, https://osf.io/78z3d/). One hundred nineteen volunteers (*M_age_* = 22.38 years, *SD_age_* = 5.69 years; 96 identifying as female, 21 identifying as male, two did not wish to say) participated in this experiment. Participants were recruited from the University of Florida (*n* = 67) and Prolific (*n* = 52; https://www.prolific.co/). (See the supplementary materials for separate demographics for each recruitment source.) The experiment was approved by the University of Florida Institutional Review Board. All participants provided informed consent.

#### Procedure and design

**Figure 1:**
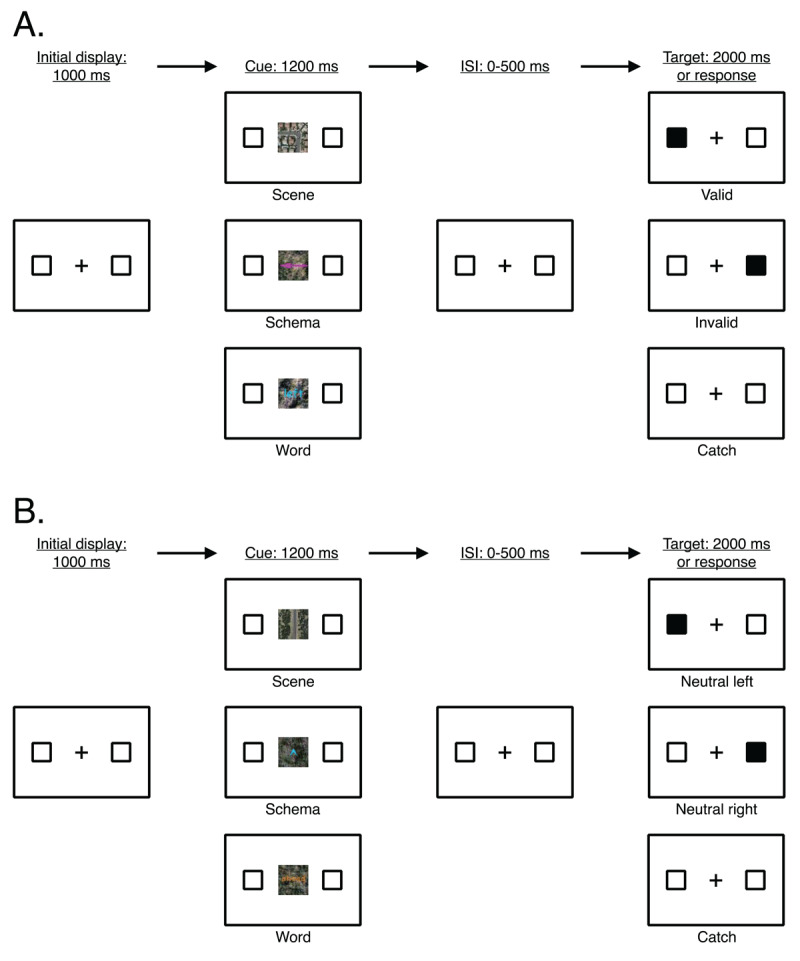
General trial structure (sequence and timing) of all trial types in Experiments 1–4. (A) Main trial types (valid, invalid, catch) defined by the direction of the cue (here, indicating left) and the location of the target. (B) Neutral trial types (neutral left, neutral right, neutral catch) defined by ahead cues. *Note*: Stimuli not drawn to scale. The target appeared for 100 ms and participants had a maximum of 2000 ms to respond. ISI = interstimulus interval.

Similar to the original spatial cueing paradigm (Posner, 1980; Posner et al., 1980), participants performed a detection task in which they responded to the presence of a black target square while RT and accuracy were recorded. The trial structure (sequence and timing) was as follows (see Figure 1): A central fixation cross was presented along with two outlined square placeholders. After 1000 ms, a cue (spatial direction) appeared at the center of the display and remained on the screen for 1200 ms. The spatial direction conveyed by the cue was equally likely to be depicted as a scene, schema, or word on any given trial. Written instructions stated that cues usually, but do not always, indicate the location where the target will appear. Left and right spatial directions indicated that an upcoming target was likely to appear on the left or right side of the screen. Ahead spatial directions were non-informative of the location of the upcoming target. Then, following a random interstimulus interval of 0–500 ms (in 100 ms intervals), a target appeared for 100 ms. Participants had 2000 ms to respond to the target by pressing the spacebar on their keyboard and were instructed to respond as quickly and accurately as possible. The primary dependent variable was RT for target present trials, which was measured from the onset of the target to when the participant pressed the spacebar. The next trial began following a randomly selected intertrial interval of 400 ms, 600 ms, or 800 ms.

Catch trials ensured selective responding to trials in which a target appeared. On catch trials, the procedure was the same except no target appeared and participants were instructed not to respond. Additionally, attention check trials were added to ensure participants were continuously engaged throughout the duration of the experiment. On attention check trials, a single digit appeared at the center of the screen for 500 ms. Participants were instructed to indicate whether the digit was even (by pressing ‘e’ on their keyboard) or odd (by pressing ‘o’ on their keyboard). The experiment did not advance until the participant gave a response on the attention check trials.

The within-subjects factors manipulated in this experiment were Target Location (left, right, none), Cue Direction (ahead, left, right), and Cue Format (scene, schema, word). There were three main trial types defined by the spatial direction depicted in the cue and the location of the target (see Figure 1, Panel A): (1) valid trial (target location and cue direction match; e.g., a target appears on the left side of the screen following a left cue); (2) invalid trial (target location and cue direction do not match; e.g., a target appears on the right side of the screen following a left cue); and (3) catch trial (target-absent; e.g., no target appears following a left cue). There were 120 experimental trials (60 trials with a left cue and 60 trials with a right cue), of which 84 were valid trials (70%), 18 were invalid trials (15%), and 18 were catch trials (15%).

Additionally, ahead cues defined a separate set of trial types (see Figure 1, Panel B). Since the target could appear either on the left or right side of the screen, the ahead spatial direction did not serve to guide space-based attention to the particular side of the screen where the target would appear, thus, acting as a neutral control in our experiment. There were 57 neutral trials, with 24 neutral trials where the target appeared on the left side of the screen (42%), 24 neural trials where the target appeared on the right side of the screen (42%), and 9 neutral trials where no target appeared (16%). Altogether, participants completed 27 practice trials with three attention check trials interspersed and 177 experimental trials with six attention check trials interspersed. Trial order was randomized across participants.

#### Apparatus and stimuli

Stimulus presentation and data collection were completed in participants’ web browsers, so viewing distance, operating system, web browser, and screen resolution varied across participants. The experiment was created in PsychoPy (version 2020.2.6; Peirce et al., 2019) and administered online via Pavlovia (https://pavlovia.org/).

**Figure 2:**
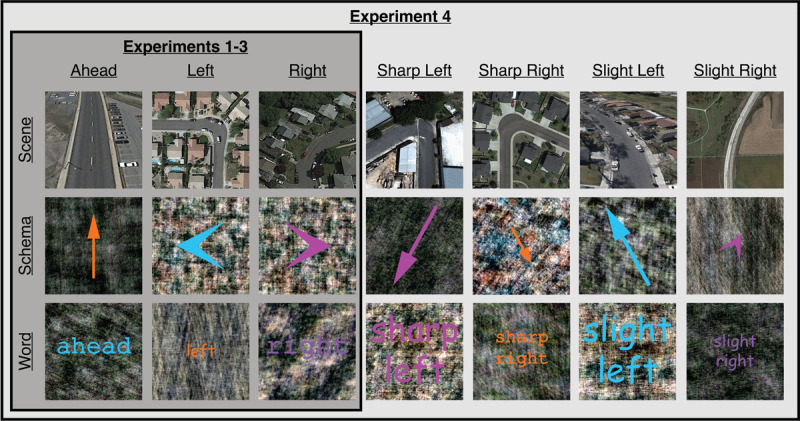
**Examples of cues used in Experiments 1–4**. In Experiments 1–3, cues depicted three orthogonal spatial directions (ahead, left, right) in three formats (scene, schema, word). In Experiment 4, non-orthogonal spatial directions (sharp left, sharp right, slight left, slight right) were included in addition to the three orthogonal spatial directions used in Experiments 1–3.

The dimensions of all stimuli described in this paper are reported in “height units”, which are specified relative to the height of the participant’s computer screen while maintaining the ratio of the height and width of the stimuli, thus ensuring that stimuli are presented consistently. For the sake of clarity, we also report the size of all stimuli in terms of pixel values based on a 13-inch (2880 × 1800 pixels) MacBook Pro computer screen. As shown in Figure 1, stimuli consisted of two black outlined square placeholders measuring .075 height units (135 pixels) wide by .075 height units (135 pixels) tall (each .006 height units, or 10 pixels, thick) located directly to the left and right of a centrally presented black fixation cross on a white screen. The vertical component of the fixation cross measured .006 height units (10 pixels) wide by .075 height units (135 pixels) tall, and the horizontal component of the fixation cross measured .075 height units (135 pixels) wide by .006 height units (10 pixels) tall. The centers of the outlined square placeholders were .378 height units (630 pixels) from the center of the fixation cross. A cue measuring .25 height units (450 pixels) wide by .25 height units (450 pixels) tall appeared at the center of the screen (see Figure 2 for examples).

Cues comprised three orthogonal spatial directions (ahead, left, right) in three formats (scenes, schemas, words). There were 20 different cues for each spatial direction and format pairing. Scenes were Google Earth overhead satellite views of roads that consisted of a single turn in a particular direction. Schemas varied in size (small, medium, large),^2^ style (arrow, chevron), and color (magenta [RGB: 255, 64, 255], orange [255, 147, 0], cyan [RGB: 0, 253, 255], purple [148, 32, 146]). Words varied in size (small, medium, large), font (Courier, Comic Sans), and color (magenta, orange, cyan, purple). Schemas and words were overlaid on phase-scrambled versions of the scenes to control for low-level image properties across representational formats. (See Weisberg and colleagues (2018) for detailed descriptions of the cues, how the cues were developed, and pilot norming tests.) The target was the filling-in of one of the outlined square placeholders to solid black. Attention check trials consisted of a single centrally presented digit (numbers 1–9; 90-point Arial font).

#### Transparency and openness

This experiment was pre-registered, including hypotheses, data collection plan, exclusion criteria, power analyses, sample sizes, and analyses. All materials, data, and experiment and analysis code, as well as pre-registration and supplementary materials, are publicly available in a repository on Open Science Framework (https://osf.io/78z3d/).

### Results

Individual participant data were checked against three pre-registered exclusion criteria. Participant data were excluded if the participant failed more than two (out of six) attention check trials (in which the participant incorrectly indicated whether a number was even or odd) or had excessively high false alarm (in which the participant responded to more than 25% of catch trials), and/or miss (in which the participant failed to respond to more than 10% of target-present trials) rates. Of the 119 participants who completed this experiment, seven were removed for failing more than two attention check trials (*M* = 4 trials, *SD* = 1 trial), leaving 112 participants who had an average false alarm rate of 2.25% (*SD* = 3.73%) and an average miss rate of 7.07% (*SD* = 22.30%). Of these 112 participants, 12 were excluded due to high miss rates (*M* = 58.22%, *SD* = 42.41%). The remaining 100 participants (*M_age_* = 22.86 years, *SD_age_* = 5.69 years; 82 identifying as female, 17 identifying as male, one did not wish to say) had an average false alarm rate of 1.93% (*SD* = 3.39%) and an average miss rate of 0.93% (*SD* = 1.54%). (See Figures S1 and S2 in the supplementary materials for histograms of false alarm and miss rates.)

Individual trial data were checked against two additional pre-registered exclusion criteria regarding our primary dependent variable. Individual trials were excluded if the RT was less than 200 ms (considered an anticipatory response) and/or was more or less than three SDs from the participant’s mean RT (considered an outlier response). In total, we discarded 84 trials (0.56% of all trials) for being anticipatory responses and 230 trials (1.54% of all trials) for being outlier responses.

#### Confirmatory results

A preliminary analysis revealed no significant main effect or interactions involving recruitment source (University of Florida and Prolific, all *F*s < 1.0), and so the data were collapsed across recruitment sources. To answer the main research questions corresponding to our three pre-registered predictions, RTs were collapsed across target location and cue direction and submitted to a 2 (trial type: valid, invalid) × 3 (cue format: scene, schema, word) within-subjects repeated measures analysis of variance (ANOVA). Mean error rates (in percentages of missed responses) are listed in Table 1 and were also submitted to the same within-subjects repeated measures ANOVA. There were no significant main effects or interactions with error rates, *F*s < 2.5, *p*s > .12, indicating no statistically significant differences across conditions. Thus, participants did not sacrifice performance for speed in this experiment and the following RT results were not influenced by error rates (critical α = .035).

**Table 1:** Error rates as a function of cue format and trial type in Experiment 1.


CUE FORMAT	*M* (*SEM*) ERROR RATE FOR VALID TRIALS	*M* (*SEM*) ERROR RATE FOR INVALID TRIALS

Scene	2.07 (1.03)	1.83 (1.23)

Schema	1.46 (1.08)	2.67 (1.29)

Word	1.54 (1.00)	2.67 (1.36)


##### Pre-registered Prediction 1: Faster performance on valid than invalid trials

We found a main effect of trial type, *F*(1,99) = 18.73, *p* < .001, η_p_^2^ = 0.16, *BF_10_* = 92,848.04 (for Bayes Factor interpretation, see Etz & Vandekerckhove, 2016; Lee & Wagenmakers, 2013; and Quintana & Williams, 2018), such that RTs were significantly faster on valid (*M* = 388.26 ms, *SEM* = 2.97 ms) than invalid (*M* = 400.34 ms, *SEM* = 3.67 ms) trials. That is, space-based attention was preferentially allocated to targets in validly cued over invalidly cued locations, reflecting a cue validity effect.

##### Pre-registered Prediction 2: Faster overall performance with schema cues than word and scene cues

We found no main effect of cue format on RTs (*M_scene_* = 393.66 ms, *SEM_scene_* = 3.93 ms; *M_schema_* = 393.34 ms, *SEM_schema_* = 4.01 ms; *M_word_* = 395.91 ms, *SEM_word_* = 4.40 ms), *F*(2,198) = 0.64, *p* = .531, *η*_p_^2^ = 0.01, *BF_10_* = 0.03 (all pairwise comparisons, *t*s < 0.9, *p*s > .35). That is, target detection speed was not influenced by the format of the central cue.

##### Pre-registered Prediction 3: Modulation of cue validity effects by cue format

The interaction between trial type and cue format was significant, *F*(2,198) = 6.52, *p* = .002, *η*_p_^2^ = 0.06, *BF_10_* = 6.60. This interaction was driven by a difference in RTs on trials with either a schema or word cue as a function of trial type. Specifically, with schema and word cues, RTs were significantly faster on valid (*M_schema_* = 385.19 ms, *SEM_schema_* = 5.18 ms; *M_word_* = 386.72 ms, *SEM_word_* = 5.16 ms) than invalid (*M_schema_* = 401.49 ms, *SEM_schema_* = 6.05 ms; *M_word_* = 405.10 ms, *SEM_word_* = 7.03 ms) trials, *t*s > 4.0, *p*s < .001, *d*s = 0.28, *BF_10_* s > 174.00. However, with scene cues, RTs were statistically equivalent on valid (*M* = 392.89 ms, *SEM* = 5.13 ms) and invalid (*M* = 394.44 ms, *SEM* = 5.98 ms) trials, *t*(99) = 0.41, *p* = .682, *d* = 0.03*, BF_10_* = 0.12. That is, significant cue validity effects were observed with schema and word cues, but not with scene cues.

**Figure 3:**
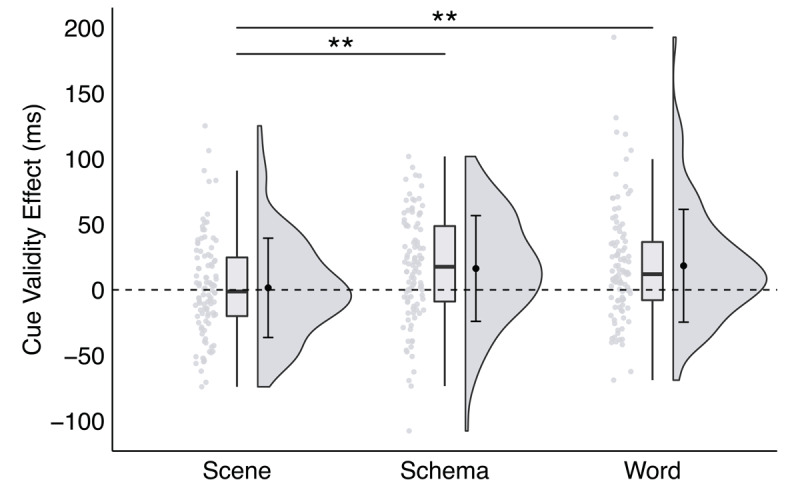
**A significant interaction between trial type and cue format in Experiment 1**, plotted as the cue validity effect as a function of cue format. *Note*: Raincloud plots were generated for each cue format (see Allen et al., 2021). Scatter plots represent individual participant cue validity effects, box plots display sample median and interquartile range, and split-half violins illustrate probability distributions of sample variances. Group mean and ±1 standard deviation of each effect is plotted inside the corresponding split-half violin. *Note*: * *p* < .035, ** *p* < .01, *** *p* < .001; critical α = .035.

To follow up, we conducted planned pairwise comparisons across those effects (see Figure 3). Specifically, the schema (*M* = 16.30 ms, *SEM* = 4.04 ms) and word (*M* = 18.38 ms, *SEM* = 4.30 ms) cue validity effects were significantly larger than the scene cue validity effect (*M* = 1.56 ms, *SEM* = 3.79 ms), *t*s > 3.1, *p*s < .002, *d*s > 0.38, *BF_10_* s > 12.00. The schema cue validity effect was not significantly different from the word cue validity effect, *t*(99) = 0.37, *p* = .709, *d* = 0.05, *BF_10_* = 0.12. This pattern of results supports our prediction and demonstrates that preferential allocation of space-based attention is greater (and more efficient) with schema and word cues than with scene cues.

See the supplementary materials for Exploratory Results.

### Discussion

The results of Experiment 1 provide support for two of our three pre-registered predictions. In support of our first prediction, participants were faster detecting a cued target on valid trials than a non-cued target on invalid trials, replicating the cue validity effect. Consistent with the original finding by Posner (1980), we interpret this result as preferential prioritization of space-based attention at the cued (valid) location compared to the non-cued (invalid) location, which provided participants with an attentional processing benefit so that targets appearing in that location were detected faster. Regarding our second prediction, we found no significant differences in target detection times between scenes, schemas, and words. Representational format of spatial direction did not affect participants’ target detection time. However, format interacted with trial type, and thus, in support of our third prediction, cue validity effects were significant for schemas and words, but not for scenes (for which RTs on valid and invalid trials were statistically equivalent). Furthermore, the cue validity effects with schemas and words were significantly larger than the cue validity effect with scenes. This pattern of findings ultimately suggests that preferential allocation of space-based attention was present and more efficient for schema and word cues but not scene cues.

These findings partially replicate observations made by others. Like Gibson and Kingstone (2006) and Weisberg and colleagues (2018), we found an advantage for schemas/arrows over other representational formats, such as scenes in our case. But our results also add support to the notion that words can provide an efficient way to communicate spatial information, a finding that is consistent with some (Hommel et al., 2001; Weisberg et al., 2018), but not others (Gibson & Kingston, 2006). In fact, we found a larger advantage for words than schemas, although this difference did not reach statistical significance. Similarly, Hommel and colleagues found an advantage for both arrows and words, but it is unclear whether the effects for arrows and words in their study differed since they did not report any descriptive statistics or comparisons. One possible explanation for these differences across studies is variations in design and task parameters. For instance, our task closely resembled the original Posner spatial cueing paradigm (i.e., two possible target locations, participants performed a detection task, 70% of trials were valid, and we contrasted valid and invalid trials to obtain the cue validity effect). In contrast, Gibson and Kingstone (2006) incorporated large deviations from the original Posner spatial cueing paradigm (i.e., four possible target locations, participants performed a discrimination task, 100% of trials were valid, and they contrasted above/below target locations and left/right target locations).

To explain why people extracted spatial direction more slowly with scene cues, Weisberg and colleagues (2018) posited that scenes may require more costly computations to decode spatial direction due to extraneous details within the scenes (i.e., automobiles, buildings). Scenes may also contain slight deviations in the spatial direction being conveyed. For instance, not all left scene cues may depict an exact 90° left turn, but all left schema and word cues contain the same information about spatial direction (i.e., “left” or an arrow pointing exactly 90° to the left; see also Klippel & Montello, 2007). Furthermore, scenes may be more visually complex than schemas and words, requiring participants to create a different spatial frame of reference by imagining the path of travel through the scene from the bottom of the image toward the top in an egocentric perspective with respect to their own body position. If participants were not establishing an egocentric spatial frame of reference and imagining the path of travel for scenes, this could have impacted their ability to use the scenes for efficient allocation of space-based attention. To test this possibility, in Experiment 2, we explicitly instructed participants to interpret scenes from an egocentric perspective and imagine traveling through the scene to determine whether interpretation of the scenes in this manner resulted in a significant cue validity effect.

## Experiment 2 – Explicit Interpretation of Scenes

Experiment 2 continued to use the spatial cueing paradigm from Experiment 1, but, like Weisberg and colleagues (2018), we explicitly instructed participants to imagine that they are traveling from the bottom of the image toward the top and to imagine the direction they would have to turn in the scene to determine whether spatial direction comprehension and preferential allocation of space-based attention in scenes was dependent on egocentric navigation. This experiment was not pre-registered, but hypotheses, data collection plan, exclusion criteria, power analyses, sample sizes, and analyses were carried over from Experiment 1, and we note in the text when deviations occurred from the pre-registration materials.

### Method

#### Participants

One hundred twenty-two volunteers (*M_age_* = 28.16 years, *SD_age_* = 4.34 years; 83 identifying as female, 37 identifying as male, two did not wish to say) participated in this experiment. Participants were recruited from Prolific and were paid an hourly rate of $10.

#### Procedure, design, apparatus, and stimuli

The procedure, design, apparatus, and stimuli for Experiment 2 were identical to those in Experiment 1, except for the following: Participants were explicitly instructed to imagine that they are traveling from the bottom of the image toward the top and imagine the direction they would have to turn in each scene. Participants were also informed that the schemas and words varied in size, style/font, and color.

### Results

Of the 122 participants who completed this experiment, five were removed for failing more than two attention check trials (*M* = 4 trials, *SD* = 2 trials), leaving 117 participants who had an average false alarm rate of 2.18% (*SD* = 5.34%) and an average miss rate of 12.50% (*SD* = 30.68%). Of these 117 participants, 16 were excluded due to high false alarm (*n* = 2; *M* = 35.18%, *SD* = 2.62%) and/or miss (*n* = 15; *M* = 88.13%, *SD* = 27.18%) rates. The remaining 101 participants (*M_age_* = 28.32 years, *SD_age_* = 4.33 years; 67 identifying as female, 32 identifying as male, two did not wish to say) had an average false alarm rate of 1.72% (*SD* = 3.25%) and an average miss rate of 1.37% (*SD* = 2.09%). (See Figures S10 and S11 in the supplementary materials.) We also discarded 50 trials (approximately 0.33% of all trials) for being anticipatory responses and 216 trials (approximately 1.45% of all trials) for being outlier responses.

#### Confirmatory results

Mean error rates (in percentages of missed responses) are listed in Table 2. There were no significant main effects or interactions with error rates, *F*s < 1.3, *p*s > .27, indicating no statistically significant differences across conditions. Aligning with the results in Experiment 1, participants did not sacrifice performance for speed in this experiment and the following RT results were not influenced by error rates (critical α = .035).

**Table 2:** Error rates as a function of cue format and trial type in Experiment 2.


CUE FORMAT	*M* (*SEM*) ERROR RATE FOR VALID TRIALS	*M* (*SEM*) ERROR RATE FOR INVALID TRIALS

Scene	3.40 (0.42)	2.31 (0.67)

Schema	2.97 (0.35)	3.47 (0.75)

Word	2.94 (0.36)	2.64 (0.61)


##### Pre-registered Prediction 1

Consistent with Experiment 1, we found a main effect of trial type, *F*(1,100) = 40.28, *p* < .001, *η*_p_^2^ = 0.29, *BF_10_* = 21,144,786, such that RTs were significantly faster on valid (*M* = 398.36 ms, *SEM* = 3.13 ms) than invalid (*M* = 410.98 ms, *SEM* = 3.50 ms) trials.

##### Pre-registered Prediction 2

Consistent with Experiment 1, we found no main effect of cue format on RTs (*M_scene_* = 403.01 ms, *SEM_scene_* = 3.86 ms; *M_schema_* = 407.18 ms, *SEM_schema_* = 4.24 ms; *M_word_* = 403.82 ms, *SEM_word_* = 4.15 ms), *F*(2,200) = 1.55, *p* = .214, *η*_p_^2^ = 0.02, *BF_10_* = 0.08 (all pairwise comparisons, *t*s < 1.6, *p*s > .12).

##### Pre-registered Prediction 3

Consistent with Experiment 1, the interaction between trial type and cue format was significant, *F*(2,200) = 7.07, *p* = .001, *η*_p_^2^ = 0.07, *BF_10_* = 10.01. Specifically, with schema and word cues, RTs were significantly faster on valid (*M_schema_* = 396.56 ms, *SEM_schema_* = 5.75 ms; *M_word_* = 397.71 ms, *SEM_word_* = 5.42 ms) than invalid (*M_schema_* = 417.79 ms, *SEM_schema_* = 6.10 ms; *M_word_* = 409.92 ms, *SEM_word_* = 6.24 ms) trials, *t*s > 4.1, *p*s < .001, *d*s > 0.20, *BF_10_* s > 297.00. However, with scene cues, RTs were statistically equivalent on valid (*M* = 400.80 ms, *SEM* = 5.11 ms) and invalid (*M* = 405.22 ms, *SEM* = 5.81 ms) trials, *t*(100) = 1.22, *p* = .226, *d* = 0.08*, BF_10_* = 0.23.

**Figure 4:**
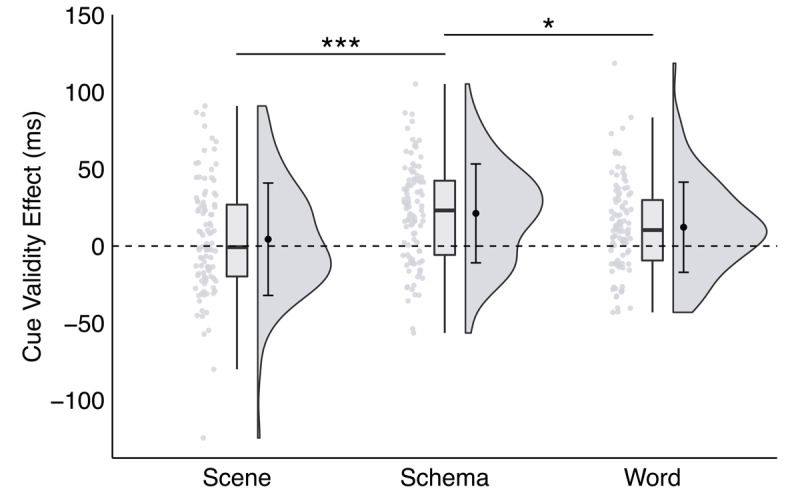
**A significant interaction between trial type and cue format in Experiment 2**, plotted as the cue validity effect as a function of cue format. *Note*: * *p* < .035, ** *p* < .01, *** *p* < .001; critical α = .035.

The schema cue validity effect (*M* = 21.23 ms, *SEM* = 3.19 ms) was significantly larger than the word (*M* = 12.22 ms, *SEM* = 2.91 ms) and scene (*M* = 4.42 ms, *SEM* = 3.63 ms) cue validity effects, *t*s > 2.3, *p*s < .02, *d*s > 0.29, *BF_10_* s > 1.6 (see Figure 4). The word cue validity effect was not significantly different from the scene cue validity effect, *t*(100) = 1.64, *p* = .105, *d* = 0.24, *BF_10_* = 0.40. That is, the explicit instruction to interpret the scenes from an egocentric perspective did not influence preferential allocation of space-based attention for scene cues.

See the supplementary materials for Exploratory Results.

### Discussion

Like Experiment 1, in Experiment 2 schemas and words successfully cued spatial attention, but scenes did not. That is, explicitly instructing participants to interpret scenes from an egocentric perspective and to imagine traveling through the scene (similar to Weisberg and colleagues, 2018) did not influence participants’ preferential allocation of space-based attention. If interpreting scenes from an egocentric perspective did not significantly modulate preferential allocation of space-based attention, why might scenes fail to influence space-based attention? A remaining explanation is that, given their visual complexity (i.e., containing elements unrelated to spatial direction) and variability across exemplars, scenes may require more processing time than schemas and words. To test this possibility, in Experiment 3, we increased the duration of the cue allowing participants more time to extract spatial direction. If failure to allocate space-based attention is one of processing time, this manipulation will lead to cue validity effects for all cue formats, including scenes.

## Experiment 3 – Cue Duration

In Experiment 3 we ensured that people had sufficient time to comprehend spatial directions in scenes to probe if under this condition space-based attention would be preferentially allocated. We continued to use the spatial cueing paradigm from Experiments 1 and 2 but varied the duration the cue was visible. This modification was done to test whether significant cue validity effects emerged with sufficient time to compute the spatial direction of the cue even for more complex stimuli like scenes. For scene cues, we expected to find a significant cue validity effect when the cue was presented for 2400 ms compared to 600 ms or 1200 ms. This would support the notion that people require additional time to comprehend the spatial direction portrayed in scenes before preferentially allocating space-based attention. This experiment was not pre-registered, but hypotheses, data collection plan, exclusion criteria, power analyses, sample sizes, and analyses were carried over from Experiments 1 and 2, and we note in the text when deviations occurred from the pre-registration materials.

### Method

#### Participants

One hundred forty-five volunteers (*M_age_* = 19.01 years, *SD_age_* = 1.47 years; 89 identifying as female, 54 identifying as male, two did not wish to say) from the University of Florida participated in this experiment in exchange for course credit.

#### Procedure, design, apparatus, and stimuli

Procedure, design, apparatus, and stimuli for Experiment 3 were identical to those in Experiment 1, except for the following: (1) We added Cue Duration (600 ms, 1200 ms, 2400 ms) as a new within-subjects factor. (2) Trials were blocked by cue duration and blocks of trials were randomly ordered for each participant. (3) Participants completed three blocks of practice trials, each with 27 trials and three attention check trials, and three blocks of experimental trials, each with 177 trials and six attention check trials. Numbers of experimental trials and attention check trials per block were the same as in Experiment 1, but, in total, participants completed three times as many trials as in Experiment 1 (corresponding to three different cue durations).

### Results

Like in Experiment 1, participants were excluded for having excessively high false alarm and/or miss rates. Participants were also excluded based on their responses to attention check trials. But to account for the increase in trials between Experiments 1 and 3, we increased this exclusion criteria as failing more than six (out of 18) attention check trials. Of the 145 participants who completed this experiment, eight were removed for failing more than six attention check trials (*M* = 8 trials, *SD* = 1 trial), leaving 137 participants who had an average false alarm rate of 5.89% (*SD* = 8.87%) and an average miss rate of 18.42% (*SD* = 33.91%). Of these 137 participants, 36 were excluded due to high false alarm (*n* = 6; *M* = 38.07%, *SD* = 11.62%) and/or miss (*n* = 34; *M* = 69.10%, *SD* = 34.73%) rates. The remaining 101 participants (*M_age_* = 19.19 years, *SD_age_* = 1.59 years; 64 identifying as female, 35 identifying as male, two did not wish to say) had an average false alarm rate of 4.29% (*SD* = 5.07%) and an average miss rate of 1.64% (*SD* = 1.93%). (See Figures S13 and S14 in the supplementary materials.) We also discarded 615 trials (1.38% of all trials) for being anticipatory responses and 707 trials (1.60% of all trials) for being outlier responses.

#### Cue duration results

To examine the effect of cue duration, RTs were submitted to a 2 (trial type: valid, invalid) × 3 (cue format: scene, schema, word) × 3 (cue duration: 600 ms, 1200 ms, 2400 ms) within-subjects repeated measures ANOVA. Mean error rates (in percentages of missed responses) are listed in Table 3. There were no significant main effects or interactions with error rates, *F*s < 2.7, *p*s > .07. Once again, aligning with the results in Experiments 1 and 2, participants did not sacrifice performance for speed in this experiment and the following RT results were not influenced by error rates (critical α = .035).

**Table 3:** Error rates as a function of cue format, cue duration, and trial type in Experiment 3.


CUE FORMAT	CUE DURATION	*M* (*SEM*) ERROR RATE FOR VALID TRIALS	*M* (*SEM*) ERROR RATE FOR INVALID TRIALS

Scene	600 ms	4.42 (0.52)	3.14 (0.80)

	1200 ms	4.39 (0.55)	4.46 (0.84)

	2400 ms	4.92 (0.50)	5.28 (0.88)

Schema	600 ms	5.23 (0.52)	4.62 (0.78)

	1200 ms	4.42 (0.58)	6.27 (0.96)

	2400 ms	4.70 (0.52)	4.13 (0.86)

Word	600 ms	4.53 (0.53)	3.63 (0.76)

	1200 ms	4.35 (0.62)	5.78 (1.03)

	2400 ms	4.56 (0.52)	5.28 (0.88)


We found a main effect of cue duration on RTs, *F*(2,200) = 9.57, *p* < .001, *η*_p_^2^ = 0.09, *BF_10_* = 20,404,918, indicating that target detection speed varied as a function of cue duration. Compared to RTs when the cue was presented for 2400 ms (*M* = 389.85 ms, *SEM* = 2.10 ms), RTs were significantly faster when the cue was presented for 600 ms (*M* = 378.02 ms, *SEM* = 2.00 ms) and 1200 ms (*M* = 383.09 ms, *SEM* = 2.14 ms), *t*s > 2.6, *p*s < .009, *d*s > 0.15, *BF_10_* s > 3.10. RTs were statistically equivalent when the cue was presented for 600 ms or 1200 ms, *t*(100) = 1.90, *p* = .061, *d* = 0.11, *BF_10_* = 0.62.

The interactions between cue format and cue duration, trial type and cue duration, and trial type, cue format, and cue duration were not significant (*p*s > .35; See the supplementary materials for additional cue duration results).

#### Confirmatory results

To answer the main research questions corresponding to our three pre-registered predictions from Experiment 1, we continue to report the results of the 2 (trial type) × 3 (cue format) × 3 (cue duration) within-subjects repeated measures ANOVA.

##### Pre-registered Prediction 1

Consistent with Experiments 1 and 2, we found a main effect of trial type, *F*(1,100) = 27.75, *p* < .001, *η*_p_^2^ = 0.22, *BF_10_* = 1,357.24, such that RTs were significantly faster on valid (*M* = 380.35 ms, *SEM* = 1.56 ms) than invalid (*M* = 386.96 ms, *SEM* = 1.84 ms) trials.

##### Pre-registered Prediction 2

Consistent with Experiments 1 and 2, we found no main effect of cue format on RTs (*M_scene_* = 382.92 ms, *SEM_scene_* = 2.01 ms; *M_schema_* = 383.46 ms, *SEM_schema_* = 2.10 ms; *M_word_* = 384.59 ms, *SEM_word_* = 2.16 ms), *F*(2,200) = 0.54, *p* = .582, *η*_p_^2^ = 0.01, *BF_10_* = 0.01 (all pairwise comparisons, *t*s < 1.0, *p*s > .35).

##### Pre-registered Prediction 3

Consistent with Experiments 1 and 2, the interaction between trial type and cue format was significant, *F*(2,200) = 12.32, *p* < .001, *η*_p_^2^ = 0.11, *BF_10_* = 240.37. This interaction was driven by a difference in RTs on trials with either a schema or word cue as a function of trial type. Specifically, for trials with a schema or word cue, RTs were significantly faster on valid (*M_schema_* = 375.96 ms, *SEM_schema_* = 2.69 ms; *M_word_* = 381.66 ms, *SEM_word_* = 2.73 ms) than invalid (*M_schema_* = 390.95 ms, *SEM_schema_* = 3.17 ms; *M_word_* = 387.52 ms, *SEM_word_* = 3.35 ms) trials, *t*s > 2.6, *p*s < .01, *d*s > 0.13, *BF_10_* s > 1.50. However, like in Experiments 1 and 2, for scene cues, RTs were statistically equivalent on valid (*M* = 383.43 ms, *SEM* = 2.67 ms) and invalid (*M* = 382.40 ms, *SEM* = 3.00 ms) trials, *t*(100) = 0.47, *p* = .641, *d* = 0.02, *BF_10_* = 0.07.

**Figure 5:**
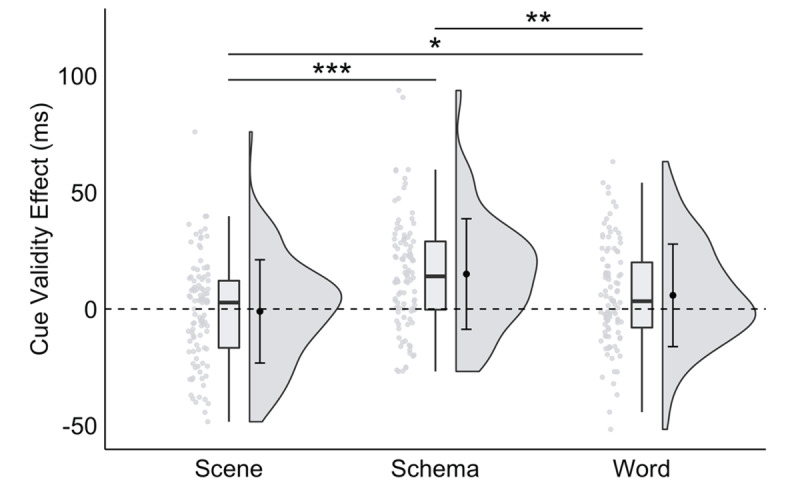
**A significant interaction between trial type and cue format in Experiment 3**, plotted as the cue validity effect as a function of cue format. *Note*: * p < .035, ** p < .01, *** p < .001; critical α = .035.

The schema cue validity effect (*M* = 14.99 ms, *SEM* = 2.30 ms) was significantly larger than the scene (*M* = -1.03 ms, *SEM* = 2.13 ms) and word (*M* = 5.86 ms, *SEM* = 2.31 ms) cue validity effects, *t*s > 2.6, *p*s < .01, *d*s > 0.40, *BF_10_* s > 3.10 (see Figure 5). Additionally, the word cue validity effect was significantly larger than the scene cue validity effect, *t*(100) = 2.32, *p* = .022, *d* = 0.31, *BF_10_* = 0.70.

See the supplementary materials for Exploratory Results.

### Discussion

In general, manipulating the duration of the cue did not significantly modulate the cue validity effect across the cue formats. In particular, significant cue validity effects emerged for all three cue durations (see Figure S15 in the supplementary materials), with the magnitude of these effects not statistically different. Furthermore, the non-significant three-way interaction indicates that there were no differences between cue validity effects across cue duration and cue format. The JZS Bayes factor for this interaction (*BF_10_* = 0.02) provided very strong evidence in support of the null hypothesis.

We replicated the findings of our pre-registered predictions from Experiments 1 and 2, but most critically, cue validity effects still did not emerge for scenes, even at longer cue durations (see Table S7 in the supplementary materials). One possibility is that scenes require even more time for the extraction of spatial direction and allocation of space-based attention due to their visual complexity; so, a significant cue validity effect may emerge with a much longer cue duration. This may not be a realistic strategy, however, when it comes to using scene cues in real-world spatial navigation in which navigation decisions must be made promptly. Even though scenes can be interpreted as lefts or rights within 1–2 seconds (Weisberg et al., 2018), the results of this experiment showed that decreasing or increasing the cue duration had no effect on the allocation of participants’ space-based attention using scenes.

## Experiment 4 – Direction Angle

Experiments 1–3 showed that space-based attention was preferentially allocated more efficiently for schema and word cues than scene cues when the cues depicted spatial directions that precisely matched the two possible target locations. Klippel and Montello (2007) found that people freely sort spatial directions based on an egocentric perspective into four orthogonal (with respect to the vertical visual field midline) spatial directions – behind (0°), right (90°), ahead (180°), and left (270°) – and four non-orthogonal spatial directions – sharp right (45°), slight right (135°), slight left (225°), and sharp left (315°). Building on this finding, we modified the spatial cueing paradigm from Experiment 1 to incorporate four non-orthogonal spatial directions in Experiment 4. Our aim was to assess the magnitude of the cue validity effect when the spatial direction conveyed by the cue and the location of the target either precisely matched (e.g., a left cue and a target on the left) or not (e.g., a sharp left cue and a target on the left).

On the one hand, space-based attention may have no effect on distinguishing between orthogonal and non-orthogonal spatial directions given our task parameters. For instance, space-based attention mechanisms may group spatial directions between 45° and 135° as all “right” directions. Thus, the speed at which people comprehend spatial directions across this range would be comparable. If that is the case, then there would be an advantage for targets on the right whenever the spatial direction of the cue depicts right, sharp right, or slight right. This outcome would suggest that space-based attention is allocated *generally* to a cued side rather than to a specific target location. On the other hand, grouping spatial directions into two broad categories (left and right) does not match the natural eight categories of spatial directions reported in Klippel and Montello (2007). People may comprehend spatial directions more slowly for non-orthogonal spatial directions (sharp right, slight right, slight left, and sharp right), which may engage space-based attention less strongly. This outcome would suggest people comprehend some spatial directions faster than others; and space-based attention is preferentially allocated more efficiently when there is a *precise* match between the spatial direction conveyed by the cue and the location of the target. Experiment 4 tested these two possibilities.

This experiment was not pre-registered, but hypotheses, data collection plan, exclusion criteria, power analyses, sample sizes, and analyses were carried over from Experiment 1. We note in the text when deviations occurred from the pre-registration materials.

### Method

#### Participants

One hundred seventeen volunteers (*M_age_* = 27.84 years, *SD_age_* = 4.81 years; 81 identifying as female, 34 identifying as male, two did not wish to say) participated in this experiment. Participants were recruited from Prolific and were paid an hourly rate of $7.25.

#### Procedure, design, apparatus, and stimuli

The procedure, design, apparatus, and stimuli for Experiment 4 were identical to those in Experiment 1, except for the following three changes: (1) The number of directions in the Cue Direction within-subjects factor increased from three to seven (ahead, left, right, sharp left, sharp right, slight left, slight right; see Figure 2 for examples). (2) We added the within-subjects factor of Direction Angle (orthogonal, sharp, slight) based on the vertical screen midline. Each level of the Direction Angle factor had two corresponding spatial directions. Left and right comprised “orthogonal”, sharp left and sharp right comprised “sharp”, and slight left and slight right comprised “slight”. (3) Participants completed three blocks of practice trials, each with 21 trials and three attention check trials, and three blocks of experimental trials, each with 139 trials and six attention check trials.

### Results

Of the 117 participants who completed this experiment, two were removed for failing more than six attention check trials (*M* = 9 trials, *SD* = 1 trial), leaving 115 participants who had an average false alarm rate of 1.68% (*SD* = 2.19%) and an average miss rate of 9.94% (*SD* = 27.53%). Of these 115 participants, 14 were excluded due to high miss rates (*M* = 74.11%, *SD* = 39.73%). The remaining 101 participants (*M_age_* = 27.78 years, *SD_age_* = 4.57 years; 70 identifying as female, 29 identifying as male, two did not wish to say) had an average false alarm rate of 1.48% (*SD* = 1.76%) and an average miss rate of 1.04% (*SD* = 1.41%). (See Figures S18 and S19 in the supplementary materials for histograms of false alarm and miss rates.) We also discarded 130 trials (approximately 0.37% of all trials) for being anticipatory responses and 552 trials (approximately 1.57% of all trials) for being outlier responses.

#### Direction angle results

To examine the effect of direction angle, RTs were collapsed across target location and cue direction and submitted to a 2 (trial type: valid, invalid) × 3 (cue format: scene, schema, word) × 3 (direction angle: orthogonal, sharp, slight) within-subjects repeated measures ANOVA. Mean error rates (in percentages of missed responses) are listed in Table 4. There were no significant main effects or interactions with error rates, *F*s < 2.9, *p*s > .06, indicating no statistically significant differences across conditions. Once again, participants did not sacrifice accuracy for performance in this experiment and the following RT results were not influenced by error rates (critical α = .035).

**Table 4:** Error rates as a function of cue format, cue angle, and trial type in Experiment 4.


CUE FORMAT	DIRECTION ANGLE	*M* (*SEM*) ERROR RATE FOR VALID TRIALS	*M* (*SEM*) ERROR RATE FOR INVALID TRIALS

Scene	Orthogonal	3.78 (0.38)	2.64 (0.65)

	Sharp	4.03 (0.42)	3.47 (0.75)

	Slight	3.04 (0.35)	2.31 (0.62)

Schema	Orthogonal	2.55 (0.32)	3.96 (0.91)

	Sharp	3.22 (0.38)	2.64 (0.61)

	Slight	2.90 (0.39)	2.97 (0.72)

Word	Orthogonal	2.65 (0.36)	3.14 (0.69)

	Sharp	2.72 (0.37)	3.30 (0.74)

	Slight	2.09 (0.29)	2.81 (0.71)


We found no main effect of direction angle (*M_orthogonal_* = 394.83 ms, *SEM_orthogonal_* = 2.55 ms; *M_sharp_* = 394.36 ms, *SEM_sharp_* = 2.51 ms; *M_slight_* = 394.36 ms, *SEM_slight_* = 2.48 ms), *F*(2,200) = 0.08, *p* = .923, *η*_p_^2^ = 0.001, *BF_10_* = 0.007 (all pairwise comparisons, *t*s < 0.4, *p*s > .70). That is, target detection speed did not vary as a function of whether the cue depicted an orthogonal or non-orthogonal direction angle (sharp or slight).

The interaction between direction angle and trial type was significant, *F*(2,200) = 6.34, *p* = .002, *η*_p_^2^ = 0.06, *BF_10_* = 3.00. This interaction was driven by a difference in RTs on trials with either orthogonal or sharp direction angles as a function of trial type. Specifically, for trials with an orthogonal direction angle (either left or right), RTs were significantly faster on valid (*M* = 388.76 ms, *SEM* = 3.56 ms) than invalid (*M* = 400.90 ms, *SEM* = 3.62 ms) trials, *t*(100) = 4.87, *p* < .001, *d* = 0.20, *BF_10_* = 50,312.45. This result is consistent with our findings from Experiments 1–3. Similarly, for trials with a sharp direction angle, RTs were significantly faster on valid (*M* = 391.90 ms, *SEM* = 3.44 ms) than invalid (*M* = 396.82 ms, *SEM* = 3.65 ms) trials, *t*(100) = 2.30, *p* = .024, *d* = 0.08, *BF_10_* = 1.65. Although the effect with sharp direction angles was statistically weaker than the effect with orthogonal direction angles, this finding provides evidence that spatial direction is comprehended equally fast for orthogonal and sharp spatial directions and that, given our task parameters, space-based attention was generally allocated to the cued side rather than to the specific target location. For trials with a slight direction angle, however, RTs were statistically equivalent on valid (*M* = 392.72 ms, *SEM* = 3.49 ms) and invalid (*M* = 396.01 ms, *SEM* = 3.53 ms) trials, *t*(100) = 1.61, *p* = .112, *d* = 0.06, *BF_10_* = 0.26. That is, significant cue validity effects were observed for orthogonal and sharp spatial directions, but not for slight spatial directions.

**Figure 6:**
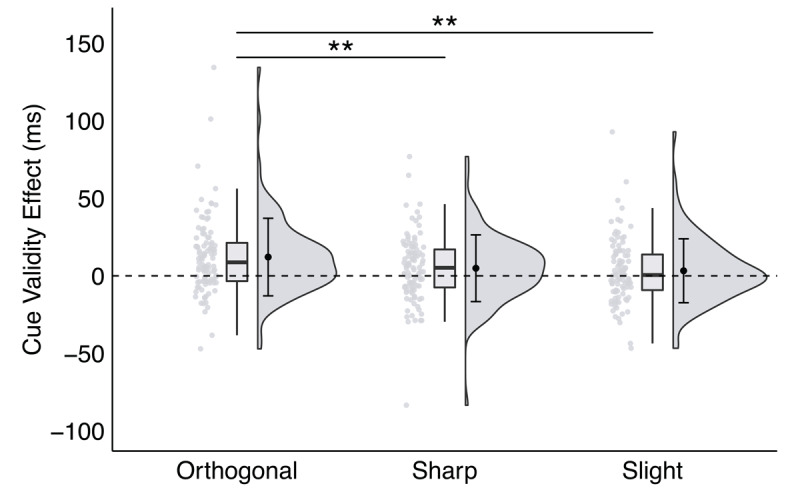
**A significant interaction between trial type and direction angle in Experiment 4**, plotted as the cue validity effect as a function of direction angle. *Note*: * p < .035, ** p < .01, *** p < .001; critical α = .035.

The orthogonal cue validity effect (*M* 12.14 ms, *SEM* = 2.27 ms) was significantly larger than the sharp (*M* = 4.92 ms, *SEM* = 1.91 ms) and slight (*M* = 3.29 ms, *SEM* = 1.95 ms) cue validity effects, *t*s > 2.60, *p*s < .01, *d*s > 0.31, *BF_10_* s > 3.10 (see Figure 6). However, the sharp cue validity effect was not significantly different than the slight cue validity effect, *t*(100) = 0.63, *p* = .528, *d* = 0.08, *BF_10_* = 0.13. While significant cue validity effects were observed for both orthogonal and sharp direction angles, the degree to which space-based attention was preferentially allocated to validly cued locations over invalidly cued locations was significantly greater for orthogonal spatial directions than for sharp and slight spatial directions.

The interactions between cue format and direction angle and trial type, cue format, and direction angle were not significant (*p*s > .39; See the supplementary materials for additional direction angle results).

#### Confirmatory results

To answer the main research questions corresponding to our three pre-registered predictions from Experiment 1, we continue to report the results of the 2 (trial type) × 3 (cue format) × 3 (direction angle) within-subjects repeated measures ANOVA.

##### Pre-registered Prediction 1

Consistent with Experiments 1–3, we found a main effect of trial type, *F*(1,100) = 17.27, *p* < .001, *η*_p_^2^ = 0.15, *BF_10_* = 1,372,420, such that RTs were significantly faster on valid (*M* = 391.13 ms, *SEM* = 2.02 ms) than invalid (*M* = 397.91 ms, *SEM* = 2.08 ms) trials.

##### Pre-registered Prediction 2

Inconsistent with Experiments 1–3, but in support of our second pre-registered prediction, we found a main effect of cue format on RTs, *F*(2,200) = 8.07, *p* < .001, *η*_p_^2^ = 0.08, *BF_10_* = 6.58. Specifically, RTs for schema (*M* = 393.84 ms, *SEM* = 2.52 ms) and word (*M* = 392.27 ms, *SEM* = 2.46 ms) cues were significantly faster than RTs for scene cues (*M* = 397.44 ms, *SEM* = 2.55 ms), *t*s > 2.7, *p*s < .01, *d*s > 0.06, *BF_10_* s > 3.40, but RTs for schema cues were statistically equivalent to RTs for word cues, *t*(100) = 1.31, *p* = .194, *d* = 0.03, *BF_10_* = 0.25. Unlike in Experiment 1–3, target detection speed was faster with schemas and words, but not scenes.

##### Pre-registered Prediction 3

Inconsistent with Experiments 1–3, and not in support of our third pre-registered prediction, the interaction between trial type and cue format was not significant, *F*(2,200) = 1.28, *p* = .280, *η*_p_^2^ = 0.01, *BF_10_* = 0.04. In short, significant cue validity effects emerged for all three cue formats which were not significantly different from each other (see Figure S21 in the supplementary materials), indicating that space-based attention was preferentially allocated with equal efficiency for all cue formats.

See the supplementary materials for Exploratory Results.

### Discussion

In this experiment, the interaction between direction angle and trial type revealed that space-based attention was preferentially allocated to validly cued locations over invalidly cued locations for cues depicting orthogonal spatial directions (i.e., left and right) as well as some non-orthogonal spatial directions (i.e., sharp left and sharp right). This finding provides evidence of significant cue validity effects for orthogonal and sharp spatial directions; but there was no evidence of significant cue validity effects for slight spatial directions. It is interesting that cue validity effects emerged for sharp spatial directions but not for slight spatial directions considering that both categories of non-orthogonal spatial directions did not match the two possible target locations. This may be due to the representations of the slight spatial directions in the present study being too similar to the representation of the ahead spatial direction (see also Klippel & Montello, 2007), which guided space-based attention equally to the left and right. In other words, space-based attention mechanisms may have treated these three spatial directions (slight left, ahead, and slight right) similarly or muddled the boundaries between these categories. In this case, cues that direct participants’ space-based attention to a location in *front* of participants, which is true for these spatial directions, resulted in the equal allocation of space-based attention to the left and right despite people categorizing slight left, ahead, and slight right as distinct spatial directions.

Also, not only did we see significant cue validity effects for both orthogonal and sharp directions, but the cue validity effect for orthogonal spatial directions was significantly larger than the cue validity effects for both kinds of non-orthogonal spatial directions. This indicates that preferential allocation of space-based attention was more efficient when the direction conveyed by the cue and the location of the target precisely matched (e.g., a target appeared on the left following a left cue) than when the direction conveyed by the cue and the location of the target did not match (e.g., a target appeared on the right following a sharp-right cue).

Despite this difference, participants were still faster to detect a target at a validly cued than an invalidly cued location, replicating findings in Experiments 1–3 and supporting our first pre-registered hypothesis. In support of our second pre-registered hypothesis (unlike Experiments 1–3), there was a main effect of cue format such that RTs to detect a target were significantly faster for schemas and words than scenes. However, regarding our third pre-registered hypothesis, trial type and cue format did not interact in Experiment 4. Rather, small, but significant, cue validity effects were observed for all three cue formats, but there were no significant differences in the magnitude of these effects across cue formats.

## General Discussion

In the present study, we tested the hypothesis that allocation of space-based attention is guided by efficient comprehension of spatial direction. Across four experiments, we consistently found a processing advantage for schemas and, less so, words in a spatial cueing task (Posner, 1980; Posner et al., 1980). No such advantage was observed for scenes, for the most part, even when participants were explicitly instructed to interpret the scene from an egocentric perspective and to imagine the path of travel (see Weisberg et al., 2018) or were given extended time to view the cues. This result is consistent with literature showing that people comprehend spatial direction faster for schemas and words than scenes (Weisberg et al., 2018). Furthermore, this result extends previous findings showing that spatially non-predictive schema and word cues bias space-based attention (Hommel et al., 2001; cf. Gibson & Kingstone, 2006). Taken together, the results support our hypothesis that schemas and words efficiently engage space-based attention, constituting a cognitive mechanism by which rapid comprehension of spatial direction underlies the allocation of space-based attention. Thus, our results suggest that overlearned communicative symbols, like arrows, and verbal directions may be more effective supports than map-like representations of turns for successful spatial navigation performance in real-world settings.

The findings from this study point to a hierarchical framework related to our hypothesis – that allocation of space-based attention is supported by efficient spatial direction comprehension for cues that match a prepotent representational structure. The strength of that association is based on the format of the cue and the angle of the spatial direction depicted in the cue. Space-based attention is most efficiently allocated for schemas and, less so, words (see Experiments 1–3) as well as cues that depict orthogonal spatial directions, like left and right (see Experiment 4), and is least efficiently allocated for cues that are visually complex, like scenes, or cues that depict non-orthogonal directions, like slight left or slight right. Such a framework echoes work by Hommel and colleagues (2001) who found that space-based attention was preferentially allocated to different spatial locations following spatially non-predictive central arrow and word cues, despite being uninformative of the location of the upcoming target. Thus, regardless of being spatially predictive or non-predictive, there appears to be a preconceived idea about what a cue should be (i.e., format, angle) and how space-based attention mechanisms should be allocated.

To our knowledge, this is the first study that used scenes, like the ones developed for Weisberg and colleagues (2018), in the Posner spatial cueing task. We show that scenes did not elicit a significant cue validity effect across experiments here (Note: a significant cue validity for scenes emerged for orthogonal spatial directions in Experiment 4 which, however, also included non-orthogonal spatial directions). Moreover, we found that RTs to detect a target were more or less consistent across the visual field and did not vary significantly by the spatial direction depicted in scenes or the location of the target on the screen. Compared to schemas and words, scenes contain more extraneous details unrelated to spatial direction and may represent spatial directions less consistently (i.e., all slight right scene cues may not always depict the exact same turn). Nevertheless, scenes, schemas, and words are commonly used to guide spatial navigation in the real world. Why, then, was space-based attention only preferentially allocated with schema and word cues but not scene cues in Experiments 1–3? We addressed three possibilities in this work.

A first possibility is that, because scenes contain more variability and are more visually complex than schemas and words, participants did not have enough time to accurately comprehend the spatial direction depicted in scenes in the present study. Weisberg and colleagues (2018) presented spatial directions in the form of scenes, schemas, and words for one second. Participants had 2.5 seconds to indicate whether the spatial direction on the current trial was the same or different as on the previous trial. Spatial direction comprehension for scenes was the slowest, and participants took, on average, 1.27 seconds to make this same/different judgement. However, this result may not have been reflective of a *pure* measure of spatial direction comprehension per se as participants not only had to extract the spatial direction depicted in the scene, but also used working memory to remember the spatial direction from the previous trial. Thus, it can be argued that basic spatial direction comprehension (i.e., extracting the spatial direction depicted in a single image without comparing it to another spatial direction) is faster than 1.27 seconds. In Experiments 1 and 2, for example, cues were displayed for 1.2 seconds so it is likely that participants had sufficient time to comprehend the spatial direction indicated by the cue, even for scenes. Moreover, results of Experiment 3 revealed that scene cues presented for twice as long (2.4 seconds) still did not preferentially allocate space-based attention to validly cued locations. Therefore, insufficient time to extract and comprehend the intended spatial direction was not likely responsible for the absence of cue validity effects for scenes.

A second possibility is that the presence of non-orthogonal spatial directions (like in Experiment 4) allowed orthogonal spatial directions in scenes to be more interpretable as conveying left or right spatial directions. In other words, sharp and slight spatial directions may have helped to reduce the ambiguity in orthogonal spatial directions, which in turn strengthened the association between the orthogonal spatial directions and the location of the targets. This would explain why a significant cue validity effect emerged for scenes depicting orthogonal spatial directions in Experiment 4, but not for scenes depicting left and right (i.e., orthogonal) spatial directions in Experiments 1–3.

A third possibility is that despite explicit instruction in Experiment 2, participants could not interpret the scenes as turns from an egocentric perspective. Weisberg and colleagues (2018) instructed participants to imagine the spatial directions in scenes as egocentric (i.e., with respect to their own body position) because computing spatial directions from scenes requires imagining travel on the path through the scene, starting at the bottom of the image. Conversely, schemas and words consist of distinguishable spatial direction information that does not require imagining travel because computing spatial direction from schemas and words is simply based on visual identification. As such, different representational formats of spatial direction can be categorized based on whether the format requires a spatial frame of reference in which spatial direction is processed egocentrically (i.e., scenes) or not (i.e., schemas and words). If participants were unable to establish an egocentric spatial frame of reference and imagine the path of travel for scenes, then this could have impacted their ability to use the scenes to allocate space-based attention. The general lack of significant differences in RTs for scenes with and without the instruction manipulation supports this possibility and indicates that scenes had no effect on biasing participants’ attention to validly cued locations. As a result, participants may not have used the scene cue at all to allocate attention and instead were simply responding to the presence of the target without regard to the scene cue. We note that this interpretation is consistent with our general hypothesis – that scenes depicting spatial directions do not have a strong pre-existing association to either the left or right visual field.

Similarly, Gibson and Kingstone (2006) interpreted their results with respect to the presence or absence of a spatial frame of reference. On the one hand, they concluded that arrows (i.e., schemas) as well as eye-gaze and abrupt onsets did not impose a spatial frame of reference. Participants were then able to process these cues quickly and with little effort, resulting in enhanced performance on the task. On the other hand, they concluded that words, which are more complex than schemas, eye-gaze, and abrupt onsets, imposed a spatial frame of reference that specified a spatial relation between a to-be-located object (i.e., the target) in reference to an external object (i.e., the cue). This required participants to spend more time and exert more effort processing word cues because a spatial frame of reference had to be established, resulting in impaired performance on the task.

Our findings provide mixed support for the notion that words require a spatial frame of reference to be processed. In Experiment 1, cue validity effects for schemas and words were not significantly different, a finding which implies that the spatial frames of reference did not differ between schemas and words. However, in Experiments 2 and 3, the schema cue validity effect was significantly greater than the word cue validity effect, suggesting a difference between spatial frames of reference for these two conditions. Moreover, since spatial direction comprehension in scenes is based on an egocentric spatial frame of reference, this could constitute a different class of spatial relation between a target and the observer that is more costly to establish than the spatial relation between the target and the cue that is required for words.

These results have important implications for our understanding of spatial direction extraction, attentional functioning, and spatial navigation in the real word. Mainly, the results we report in this paper demonstrate efficient allocation of space-based attention is supported by enhanced spatial direction comprehension for both schemas and words. Thus, navigating in visually cluttered environments, such as deciding which on- or off-ramp to take on a busy highway or searching for the baggage claim in a crowded airport terminal, depends on our ability to allocate attention to relevant navigation cues (like schemas and words) and extract their meaning to execute a specific navigation behavior (i.e., take the next exit on the right or continue walking forward to the baggage claim). Additionally, our findings extend beyond basic science to inform clinical and translational research domains. We found an association between spatial direction comprehension and the preferential allocation of space-based attention. Mechanisms underlying these two cognitive processes are known to share common cortical structures in healthy young adults, such as the intraparietal sulcus and the superior parietal lobule (Corbetta, Kincade, Ollinger, McAvoy, & Shulman, 2000; Galati, Pelle, Berthoz, & Committeri, 2010; Hopfinger, Buonocore, & Mangun, 2000; Schindler & Bartels, 2013; Vandenberghe, Gitelman, Parrish, & Mesulam, 2001; Weisberg et al., 2018; Whitlock, Sutherland, Witter, Moser, & Moser, 2008; Yantis et al., 2002). When spatial navigation behavior declines, such as in aging and Alzheimer’s disease and related dimentias therapies and interventions aimed at preserving or enhancing spatial navigation function can build upon a more thorough understanding of the association between spatial direction comprehension and space-based attention by targeting these specific cognitive processes and their corresponding brain regions.

### Future Directions

There are several important follow-up questions and future directions for this line of work. For one, we believed that explicit instruction regarding how scenes should be interpreted would result in a significant cue validity effect; however, this manipulation did not significantly modulate the cue validity effect for scenes. Since scenes are more visually complex and contain more extraneous details than schemas and words, participants may have been unable to interpret the scenes as turns (although presenting the cues for longer to allow more processing time, as in Experiment 3, did not result in a significant cue validity effect for scenes). A deeper conceptualization of scene complexity can be explored by systematically altering certain components that make scenes difficult to interpret (e.g., varying the angle depicted within a spatial direction category, removing irrelevant visual features, simplifying the turn depicted with a basic geometric shape).

Second, direction angle in Experiment 4 may have been task-irrelevant since, for example, any cue indicating the left side of the screen (i.e., left, sharp left, slight left) signaled that the upcoming target was likely to appear in the left target placeholder. Hommel and colleagues (2001) and Gibson and Kingstone (2006) included four target placeholders in their tasks that corresponded to four spatial directions. The present work can be expanded by using a display consisting of six target placeholders to address whether space-based attention can be preferentially allocated to cued locations using non-orthogonal spatial directions that precisely match target locations. Several other studies using spatially non-predictive peripheral cues (Chen, Moore, & Mordkoff, 2008; Gawryszewski, Carreiro, & Magalhães, 2005; Prinzmetal, Ha, & Khani, 2010) and spatially predictive central arrow cues (Ceballos, Tivis, Lawton-Craddock, & Nixon, 2005; Geng & Behrmann, 2005) have varied the number of matching target locations and spatial directions between four to eight. However, these studies typically report a single mean RT (or error rate) for valid and invalid trials without accounting for the location of the target in the visual field. Because performance across the visual field is not homogenous (Anderson, Cameron, & Levine, 2014; Cameron, Tai, & Carrasco, 2002; Carrasco, Talgar, & Cameron, 2001; He, Cavanagh, & Intriligator, 1996; Talgar & Carrasco, 2002), increasing the number of target placeholders corresponding to each spatial direction would require the parcellation of the data by target location. This would result in each target placeholder being a valid location and multiple invalid locations that vary based on the angular distance between the location indicated by the cue and the location of the target (see Gawryszewski et al., 2005).

Third, scenes provide the opportunity to test the importance of decision-points and perspective on the saliency of spatial directions and how these factors influence the allocation of space-based attention. Navigation decision points (i.e., intersections) are critical for the creation of cognitive maps (Janzen, 2006) and the lack of decision points could have yielded the non-significant effects for scene cues in our study. The effect of decision points can be tested by comparing performance on our task with scene cues (overhead views) that contain a decision point (e.g., highlighting a direction at an intersection) or not. The perspective of the scene can also be manipulated by comparing attentional allocation using scenes that depict a first-person, or route, perspective display (e.g., Bonner & Epstein, 2017) and an overhead view of a turn in a road, or a survey perspective display (e.g., Weisberg et al., 2018). Brunyé, Gardony, Mahoney, and Taylor (2012) observed navigation performance differences after learning a large-scale virtual environment from route and survey perspectives, suggesting that perspective affects spatial representations. Thus, it is reasonable to expect that different perspectives would modulate the allocation of space-based attention.

## Conclusion

In sum, our findings support the idea that rapid spatial direction comprehension serves as a cognitive mechanism for allocating space-based attention. These data introduce new considerations for theories of spatial cognition and navigation, which must account for space-based attention mechanisms to understand how people represent their spatial surroundings. Furthermore, our results can be applied to real-world spatial navigation in which schemas and words facilitate successful movement through the environment.

## Data Accessibility Statement

Materials, data, and experiment and analysis code for all experiments, as well as pre-registration materials for Experiment 1, are publicly available in a repository on Open Science Framework (https://osf.io/78z3d/).

## Additional File

The additional file for this article can be found as follows:

10.5334/joc.325.s1 Supplementary Files. Additional confirmatory and exploratory analyses can be found in a repository on Open Science Framework (https://osf.io/78z3d/).

## Footnotes

To summarize, statistical power was simulated using a range of effect sizes based on previous literature (*d* = 0.1–0.6) and hypothetical sample sizes (10-300 participants). Based on simulations, sample size estimates between 60 and 120 participants should be sufficient to detect small to medium sized effects (*d* = 0.2–0.3) at 80% power and medium to large sized effects (*d* = 0.4–0.5) at 95% power. Sample size estimates between 100 and 200 participants should be sufficient to detect those effect sizes at 95% power.

All cues were square images of the same size to control for the extent of visual stimulation across cue format. The size of the arrow or chevron for schema cues and the size of the font for word cues within the square was either small, medium, or large. Schema and word cues varied in size, color, and font/style to match the variability among scene cues.
