# Supplementary material for: Allocation of Space-Based Attention is Guided by Efficient Comprehension of Spatial Direction
Source: J Cogn. 2024 Jan 8;7(1):1. doi: 10.5334/joc.325 (PMC10785961; doi:10.5334/joc.325)
Supplement: Supplementary Files. — Additional confirmatory and exploratory analyses can be found in a repository on Open Science Framework (https://osf.io/78z3d/). [file joc-7-1-325-s1.pdf]

Experiment 1 – False Alarm and Miss Rates

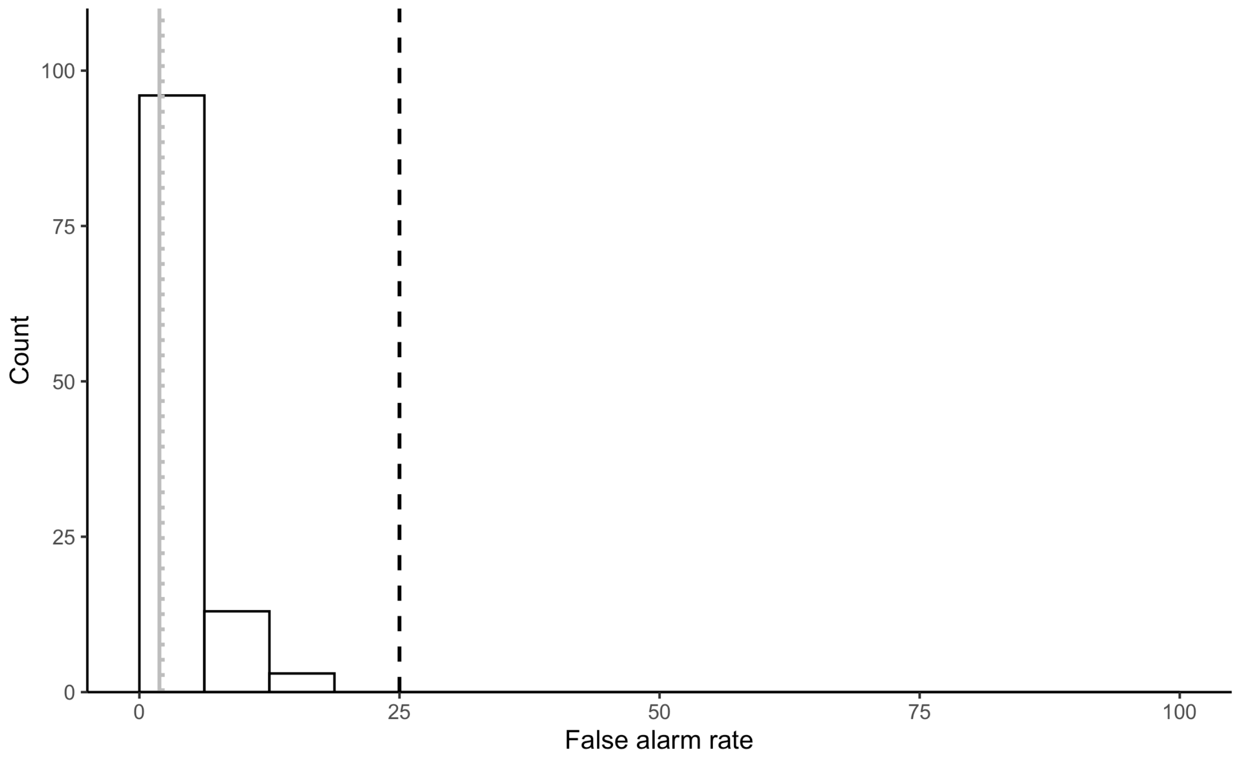

Figure S1. **Histogram of participant false alarm rates from Experiment 1.** The dashed black line at 25% marks the cutoff – participants with a false alarm rate greater than 25% were excluded. No participants had a false alarm rate greater than 25%. The dotted gray line indicates the average false alarm rate for all 112 participants (2.25%). The solid gray line indicates the average false alarm rate for the 100 included participants (1.93%).

Note. Twelve participants were excluded due to high miss rates.

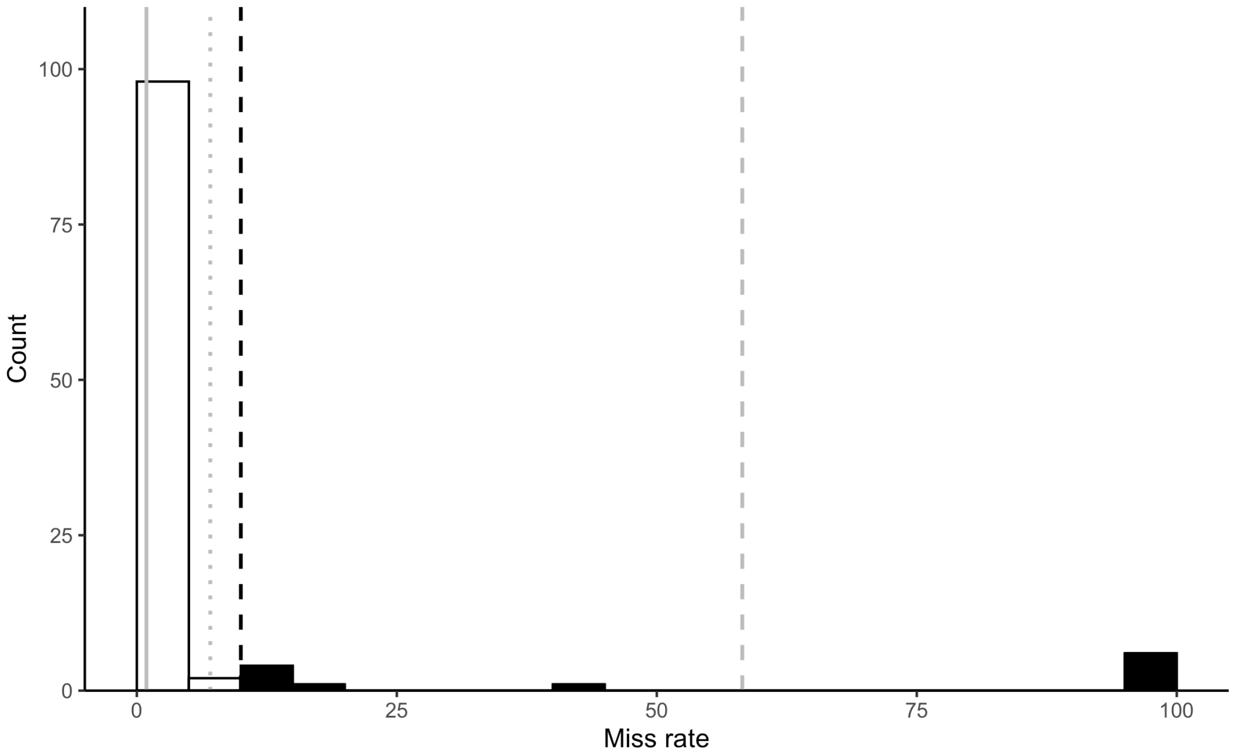

***Figure S2*. Histogram of participant miss rates from Experiment 1.** The dashed black line at 10% marks the cutoff – participants with a miss rate greater than 10% were excluded. Twelve participants had a miss rate greater than 10% (black filled bars). The dotted gray line indicates the average miss rate for all 112 participants (7.07%). The dashed gray line indicates the average miss rate for the 12 excluded participants (58.22%). The solid gray line indicates the average miss rate for the 100 included participants (0.93%). A Welch independent samples *t*-test revealed a significantly larger miss rate for the 12 excluded participants than the 100 included participants, $t(11.00) = 4.68$, $p < .001$, $d = 4.25$.

Experiment 1 – Participants recruited from the University of Florida

Participants

Sixty-seven volunteers (M_{age} = 18.49 years, SD_{age} = 1.01 years; 51 identifying as female, 14 identifying as male, two did not wish to say) at the University of Florida participated in this experiment in exchange for credit in an introductory psychology course. The experiment was approved by the University of Florida Institutional Review Board. All participants provided informed consent.

Results

Of the 67 participants who completed this experiment, six were removed for failing more than two attention check trials (M = 4 trials, SD = 1 trial), leaving 61 participants who had an average false alarm rate of 2.85% (SD = 4.19%) and an average miss rate of 12.07% (SD = 29.28%). Of these 61 participants, 11 were excluded due to high miss rates (M = 61.76%, SD = 42.59%). The remaining 50 participants (M_{age} = 18.60 years, SD_{age} = 1.12 years; 38 identifying as female, 11 identifying as male, one did not wish to say) had an average false alarm rate of 2.30% (SD = 3.73%) and an average miss rate of 1.13% (SD = 1.80%). A Welch independent samples t-test revealed a significantly larger miss rate for the 11 excluded participants than the 50 included participants, $t(10.01) = 4.72$, $p < .001$, $d = 3.44$. (See Figures S3 and S4). Additionally, we discarded 51 trials (approximately 0.67% of all trials) for being anticipatory responses and 116 trials (approximately 1.54% of all trials) for being outlier responses.

Confirmatory Results. To answer the main research questions corresponding to our three pre-registered predictions, RTs were collapsed across target location and cue direction and submitted to a 2 (trial type: valid, invalid) x 3 (cue format: scene, schema, word) within-subjects repeated measures ANOVA. Mean error rates (in percentages of missed responses) are listed in

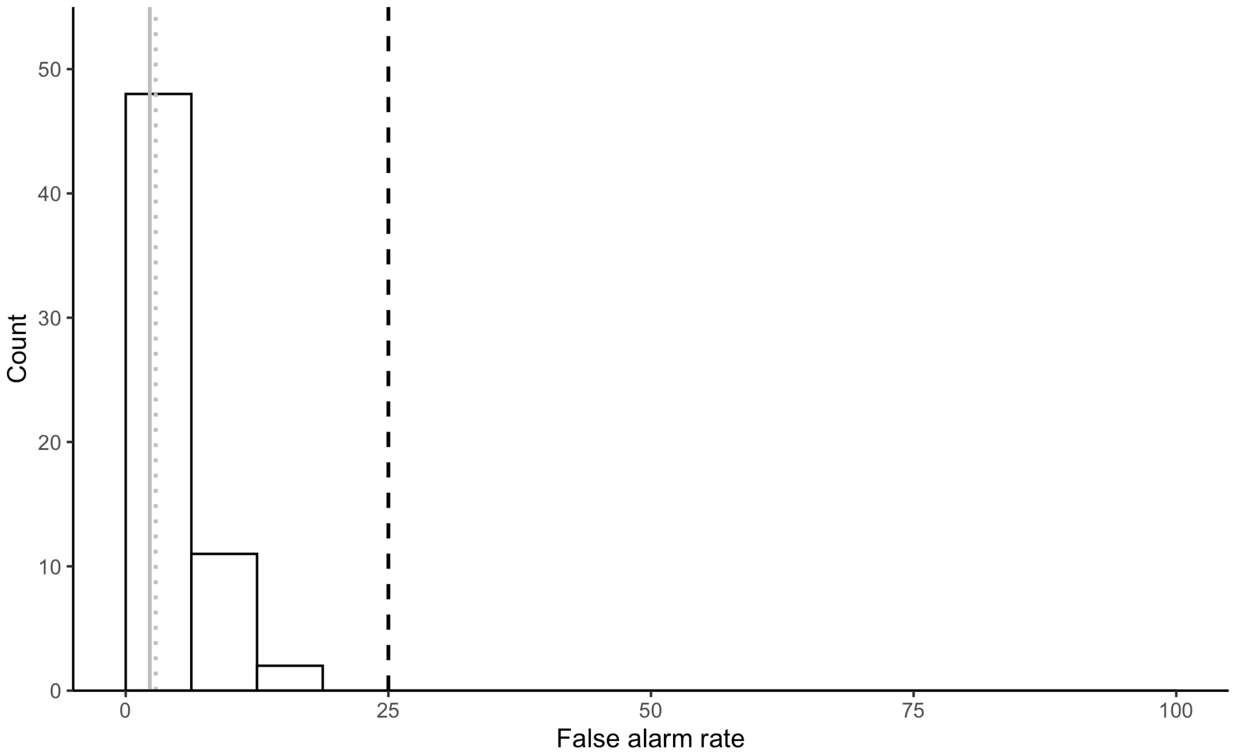

Figure S3. **Histogram of false alarm rates for participants recruited from the University of Florida in Experiment 1.** The dashed black line at 25% marks the cutoff – participants with a false alarm rate greater than 25% were excluded. Zero participants had a false alarm rate greater than 25%. The dotted gray line indicates the average false alarm rate for all 61 participants (2.85%). The solid gray line indicates the average false alarm rate for the 50 included participants (2.30%).
Note. Eleven participants were excluded due to high miss rates.

Table S1 and were also submitted to the same 2 (trial type) x 3 (cue format) repeated measures ANOVA. There were no significant main effects or interactions with error rates, Fs < 2.3, ps > .10, indicating no statistically significant differences across conditions. Thus, participants did not sacrifice accuracy for speed in this experiment, and the results reported below were not qualified by a speed-accuracy tradeoff.

Pre-registered Prediction 1: Faster performance on valid than invalid trials. We found a main effect of trial type, $F(1,49) = 8.15$, $p = .006$, $\eta_p^2 = 0.14$, $BF_{10} = 166.12$, such that RTs

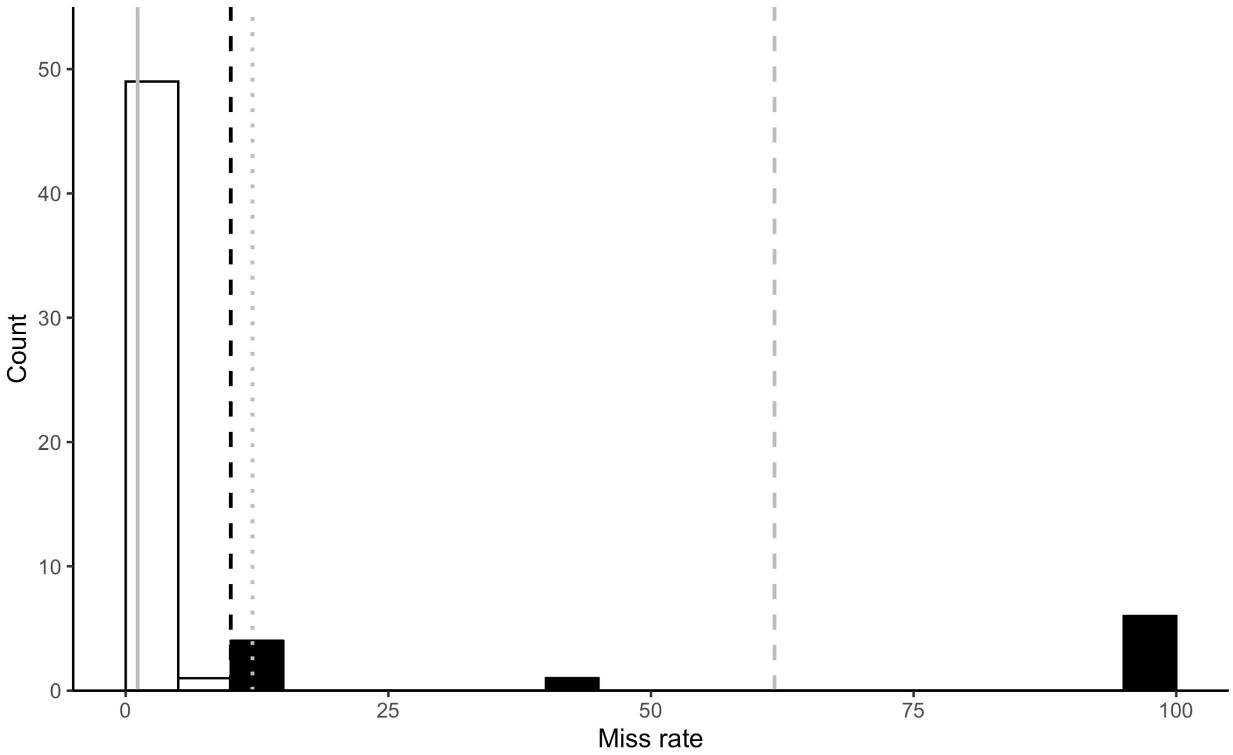

***Figure S4*. Histogram of miss rates for participants recruited from the University of Florida in Experiment 1.** The dashed black line at 10% marks the cutoff – participants with a miss rate greater than 10% were excluded. Eleven participants had a miss rate greater than 10% (black filled bars). The dotted gray line indicates the average miss rate for all 61 participants (12.07%). The dashed gray line indicates the average miss rate for the 11 excluded participants (61.76%). The solid gray line indicates the average miss rate for the 50 included participants (1.13%).

Table S1. *Error rates as a function of cue format and trial type for participants recruited from the University of Florida in Experiment 1.*

Cue Format	*M* (*SEM*) Error Rate for Valid Trials	*M* (*SEM*) Error Rate for Invalid Trials
Scene	1.86 (2.02)	0.67 (2.23)
Schema	0.93 (2.14)	0.67 (2.28)
Word	0.57 (1.96)	3.00 (2.56)

were significantly faster on valid (M = 386.91 ms, SEM = 4.25 ms) than invalid (M = 400.24 ms, SEM = 5.46 ms) trials.

Pre-registered Prediction 2: Faster overall performance with schema cues than word and scene cues. We found no main effect of cue format on RTs (M_{scene} = 391.83 ms, SEM_{scene} = 5.70 ms; M_{schema} = 392.67 ms, SEM_{schema} = 5.96 ms; M_{word} = 396.22 ms, SEM_{word} = 6.42 ms), $F(2,98) = 0.81$, $p = .447$, $\eta_p^2 = 0.02$, $BF_{10} = 0.06$ (all pairwise comparisons, *t*s < 1.2, *p*s > .26). That is, target detection speed was not influenced by the format of the central cue.

Pre-registered Prediction 3: Modulation of cue validity effects by cue format. The interaction between trial type and cue format was significant, $F(2,98) = 5.65$, $p = .005$, $\eta_p^2 =$ 0.10, $BF_{10} = 3.79$. This interaction was driven by a difference in RTs on trials with either a schema or word cue as a function of trial type. Specifically, with schema cues, RTs were significantly faster on valid (M = 384.64 ms, SEM = 7.51 ms) than invalid (M = 400.71 ms, SEM = 9.19 ms) trials, $t(49) = 2.62$, $p = .012$, $d = 0.26$, $BF_{10} = 3.26$. Similarly, with word cues, RTs were also significantly faster on valid (M = 384.45 ms, SEM = 7.42 ms) than invalid (M = 408.00 ms, SEM = 10.29 ms) trials, $t(49) = 3.57$, $p < .001$, $d = 0.34$, $BF_{10} = 34.14$. However, with scene cues, RTs were statistically equivalent on valid (M = 391.65 ms, SEM = 7.23 ms) and invalid (M = 392.02 ms, SEM = 8.90 ms) trials, $t(49) = 0.06$, $p = .949$, $d = 0.01$, $BF_{10} = 0.15$. That is, significant cue validity effects were observed with schema and word cues, but not with scene cues.

We then conducted planned pairwise comparisons across those effects (See Figure S5). Specifically, the schema cue validity effect (M = 16.06 ms, SEM = 6.14 ms) and the word cue validity effect (M = 23.55 ms, SEM = 6.60 ms) were significantly larger than the scene cue validity effect (M = 0.37 ms, SEM = 5.80 ms), $t(49) = 2.55$, $p = .014$, $d = 0.37$, $BF_{10} = 2.84$ and $t(49) = 3.34$, $p = .002$, $d = 0.53$, $BF_{10} = 18.85$, respectively. The schema cue validity effect was

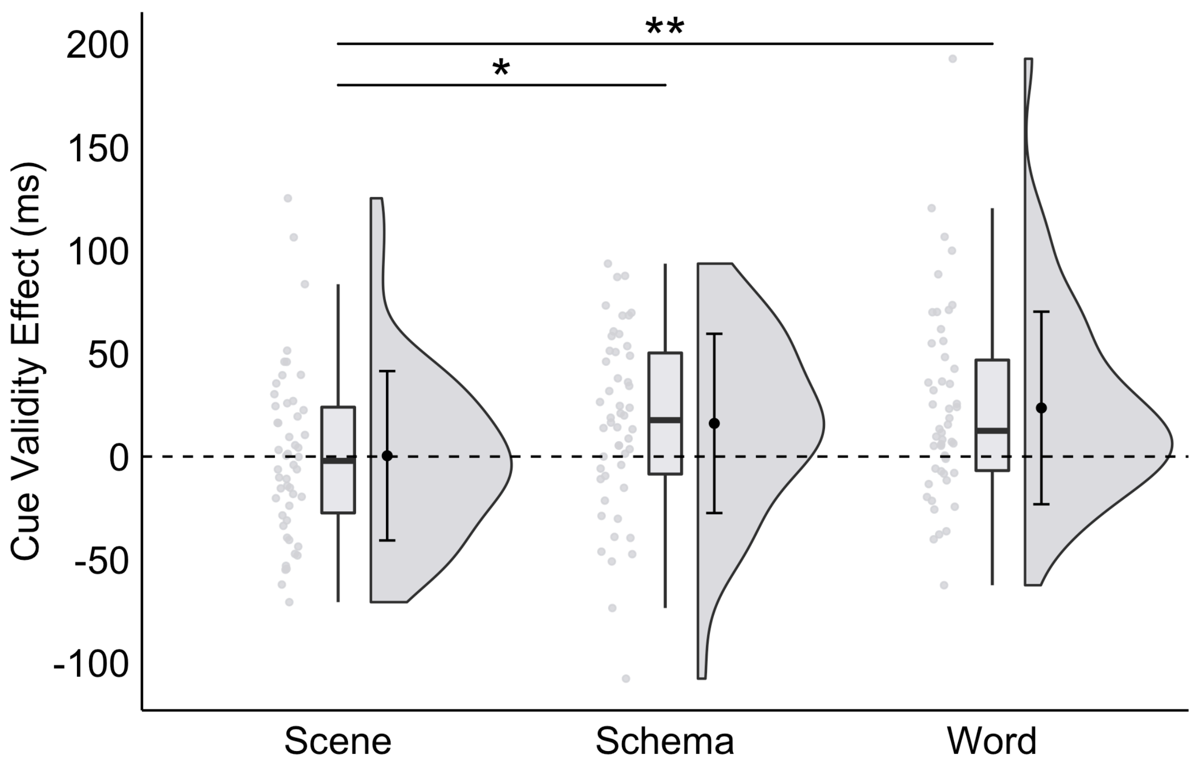

***Figure S5.* A significant interaction between trial type and cue format for participants recruited from the University of Florida in Experiment 1**, plotted as the cue validity effect as a function of cue format.
Note: Raincloud plots were generated for each cue format (see Allen et al., 2021). Scatter plots represent individual participant cue validity effects, box plots display sample median and interquartile range, and split-half violins illustrate probability distributions of sample variances. Group mean and ±1 standard deviation of each effect is plotted inside the corresponding split-half violin.
Note: * $p < .05$, ** $p < .01$, *** $p < .001$; critical $\alpha = .05$.

not significantly different than the word cue validity effect, $t(49) = 0.94$, $p = .350$, $d = 0.17$, $BF_{10} = 0.23$.

Exploratory Results. To explore differences in how attention was allocated with cues that provided spatial information of the upcoming target (i.e., left or right spatial directions) compared to cues that did not provide any spatial information of the upcoming target (i.e., ahead spatial direction; neutral trials), RTs were submitted to a 2 (target location: left, right) x 3 (cue direction: ahead, left, right) x 3 (cue format: scene, schema, word) within-subjects repeated

measures ANOVA. There were no significant main effects of target location, cue direction, or cue format, or significant interactions between target location and cue format or between cue direction and cue format, *F*s < 1.2, *p*s > .30. However, there was a significant interaction between target location and cue direction, $F(2,98) = 6.35$, $p = .003$, $\eta_p^2 = 0.12$, $BF_{10} = 551.72$, that was driven by differences in RTs on trials with either a left or right cue as a function of target location (See the upper left plot in Figure S6). In particular, for trials with a left cue, RTs were significantly faster when the target appeared on the left ($M = 385.85$ ms, $SEM = 4.19$ ms) than the right ($M = 396.96$ ms, $SEM = 5.33$ ms), $t(149) = 2.65$, $p = .009$, $d = 0.18$, $BF_{10} = 2.62$. In

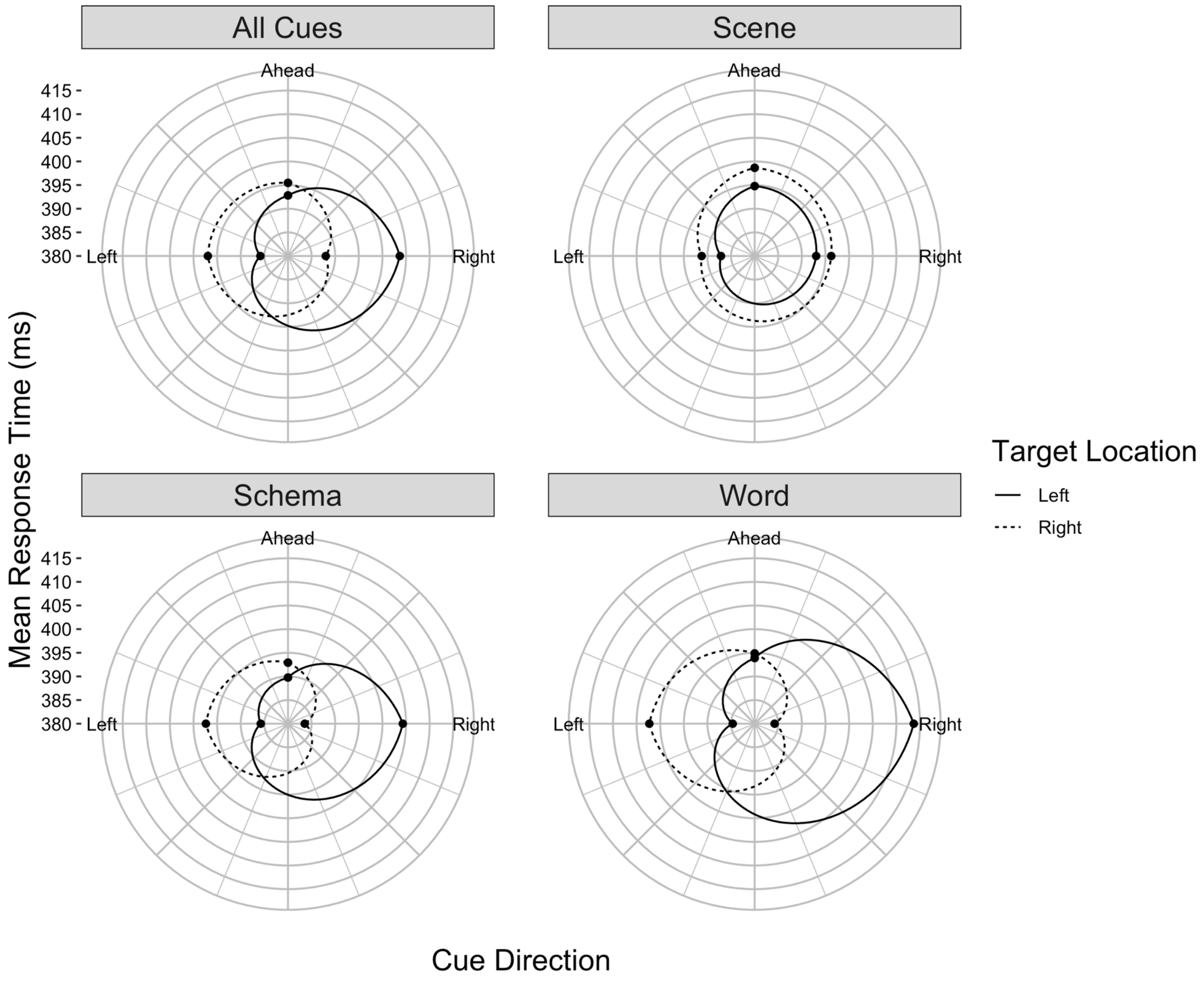

Figure S6. **Exploratory results for participants recruited from the University of Florida in Experiment 1.**

contrast, for trials with a right cue, RTs were significantly faster when the target appeared on the right (*M* = 387.97 ms, *SEM* = 4.69 ms) than the left (*M* = 403.68 ms, *SEM* = 6.76 ms), $t(149) = 3.02$, $p = .003$, $d = 0.21$, $BF_{10} = 6.96$. Critically, for trials with an ahead cue, RTs were statistically equivalent when the target appeared on the left (*M* = 392.82 ms, *SEM* = 4.97 ms) or the right (*M* = 395.48 ms, *SEM* = 4.54 ms), $t(149) = 0.81$, $p = .418$, $d = 0.05$, $BF_{10} = 0.13$.

The interaction between target location, cue direction, and cue format was also significant, $F(4,196) = 3.14$, $p = .016$, $\eta_p^2 = 0.06$, $BF_{10} = 0.91$, indicating that the interaction between target location and cue direction was further modulated by the format of the cue. Specifically, for left word cues (See the lower right plot in Figure S6), RTs were significantly faster when the target appeared on the left than the right (See Table S2). This effect was marginally significant for schema cues (See the lower left plot in Figure S6). Conversely, for right schema and word cues, RTs were significantly faster when the target appeared on the right than the left. For ahead schema and word cues, there were no differences between RTs when the target appeared on the left or the right. However, for scene cues (See the upper right plot in

Table S2. *RTs as a function of cue format, cue direction, and target location for participants recruited from the University of Florida in Experiment 1.*

Cue Format	Cue Direction	*M* (*SEM*) RT for Left Targets (ms)	*M* (*SEM*) RT for Right Targets (ms)	*t*	*p*	*d*	JZS Bayes factor (BF_{10})
	Ahead	394.79 (8.45)	398.66 (8.92)	0.62	.538	0.06	0.18
Scene	Left	387.13 (6.48)	391.22 (9.23)	0.53	.596	0.07	0.18
	Right	393.02 (10.84)	396.22 (8.66)	0.39	.697	0.04	0.17
	Ahead	389.76 (7.80)	392.92 (7.54)	0.67	.509	0.06	0.19
Schema	*Left*	*385.76 (7.66)*	*397.38 (7.89)*	*1.92*	*.061*	*0.21*	*0.83*
	**Right**	**404.32 (12.14)**	**383.55 (7.71)**	**2.26**	**.028**	**0.27**	**1.57**
	Ahead	393.90 (9.62)	394.86 (7.17)	0.16	.874	0.01	0.16
Word	**Left**	**384.65 (7.72)**	**402.30 (10.50)**	**2.21**	**.032**	**0.26**	**1.42**
	**Right**	**413.69 (12.17)**	**384.23 (8.04)**	**3.22**	**.002**	**0.38**	**13.64**

Note: Significant differences between left and right targets are bolded, and marginal differences are italicized (critical $\alpha = .05$).

Figure S6), there were no differences between RTs when the target appeared on the left or the right regardless of the spatial direction indicated by the cue.

Experiment 1 – Participants recruited from Prolific

Participants

Fifty-two volunteers (M_{age} = 27.38 years, SD_{age} = 5.30 years; 45 identifying as female, seven identifying as male) participated in this experiment. Participants were recruited from Prolific (https://www.prolific.co/) and were paid an hourly rate of $7.25.

Results

One participant was removed for failing four attention check trials, leaving 51 participants who had an average false alarm rate of 1.52% (*SD* = 2.98%) and an average miss rate of 1.08% (*SD* = 2.87%). One additional participant was excluded due to a high miss rate (19.33%). The remaining 50 participants (M_{age} = 27.12 years, SD_{age} = 5.22 years; 44 identifying as female, six identifying as male) had an average false alarm rate of 1.56% (*SD* = 3.00%) and an average miss rate of 0.72% (*SD* = 1.21%). Additionally, we discarded 33 trials (approximately 0.44% of all trials) for being anticipatory responses and 114 trials (approximately 1.54% of all trials) for being outlier responses.

Confirmatory Results. To answer the main research questions corresponding to our three pre-registered predictions, RTs were collapsed across target location and cue direction and submitted to a 2 (trial type: valid, invalid) x 3 (cue format: scene, schema, word) within-subjects ANOVA. Mean error rates (in percentages of missed responses) are listed in Table S3 and were also submitted to the same 2 (trial type) x 3 (cue format) repeated measures ANOVA. There were no significant main effects or interactions with error rates, Fs < 2.6, ps > .11, indicating no statistically significant differences across conditions. Thus, participants did not sacrifice accuracy for speed in this experiment, and the results reported below were not qualified by a speed-accuracy tradeoff.

Table S3. *Error rates as a function of cue format and trial type for participants recruited from Prolific in Experiment 1.*

Cue Format	*M* (*SEM*) Error Rate for Valid Trials	*M* (*SEM*) Error Rate for Invalid Trials
Scene	2.29 (0.41)	3.00 (1.03)
Schema	2.00 (0.34)	4.67 (1.17)
Word	2.50 (0.44)	2.33 (0.95)

Pre-registered Prediction 1. We found a main effect of trial type, $F(1,49) = 12.22$, $p = .001$, $\eta_p^2 = 0.20$, $BF_{10} = 73.86$, such that RTs were significantly faster on valid ($M = 389.61$ ms, $SEM = 4.18$ ms) than invalid ($M = 400.44$ ms, $SEM = 4.94$ ms) trials.

Pre-registered Prediction 2. We found no main effect of cue format on RTs ($M_{scene} = 395.49$ ms, $SEM_{scene} = 5.43$ ms; $M_{schema} = 394.00$ ms, $SEM_{schema} = 5.41$ ms; $M_{word} = 395.60$ ms, $SEM_{word} = 6.04$ ms), $F(2,98) = 0.14$, $p = .870$, $\eta_p^2 = 0.003$, $BF_{10} = 0.04$, all pairwise comparisons, *t*s < 0.5, *p*s > .65). That is, target detection speed was not influenced by the format of the central cue.

Pre-registered Prediction 3. The interaction between trial type and cue format was not significant, $F(2,98) = 1.93$, $p = .151$, $\eta_p^2 = 0.04$, $BF_{10} = 0.38$, but the pattern of pairwise comparisons replicates what is reported above with the participants from the University of Florida. Specifically, with schema cues, RTs were significantly faster on valid ($M = 385.73$ ms, $SEM = 7.21$ ms) than invalid ($M = 402.27$ ms, $SEM = 7.97$ ms) trials, $t(49) = 3.12$, $p = .003$, $d = 0.31$, $BF_{10} = 10.68$. Similarly, with word cues, RTs were also significantly faster on valid ($M = 388.99$ ms, $SEM = 7.23$ ms) than invalid ($M = 402.20$ ms, $SEM = 9.66$ ms) trials, $t(49) = 2.41$, $p = .020$, $d = 0.20$, $BF_{10} = 2.09$. However, with scene cues, RTs were statistically equivalent on valid ($M = 394.12$ ms, $SEM = 7.36$ ms) and invalid ($M = 396.86$ ms, $SEM = 8.06$ ms) trials, $t(49) = 0.55$, $p = .582$, $d = 0.05$, $BF_{10} = 0.18$.

We also conducted planned pairwise comparisons across those effects (See Figure S7). Specifically, the schema cue validity effect (M = 16.54 ms, SEM = 5.30 ms) was marginally larger than the scene cue validity effect (M = 2.74 ms, SEM = 4.94 ms), $t(49) = 1.97$, $p = .054$, $d = 0.38$, $BF_{10} = 0.91$. However, the word cue validity effect (M = 13.22 ms, SEM = 5.49 ms) was not significantly different than the scene cue validity effect, $t(49) = 1.47$, $p = .148$, $d = 0.28$, $BF_{10} = 0.42$, or schema cue validity effect, $t(49) = 0.42$, $p = .674$, $d = 0.09$, $BF_{10} = 0.17$.

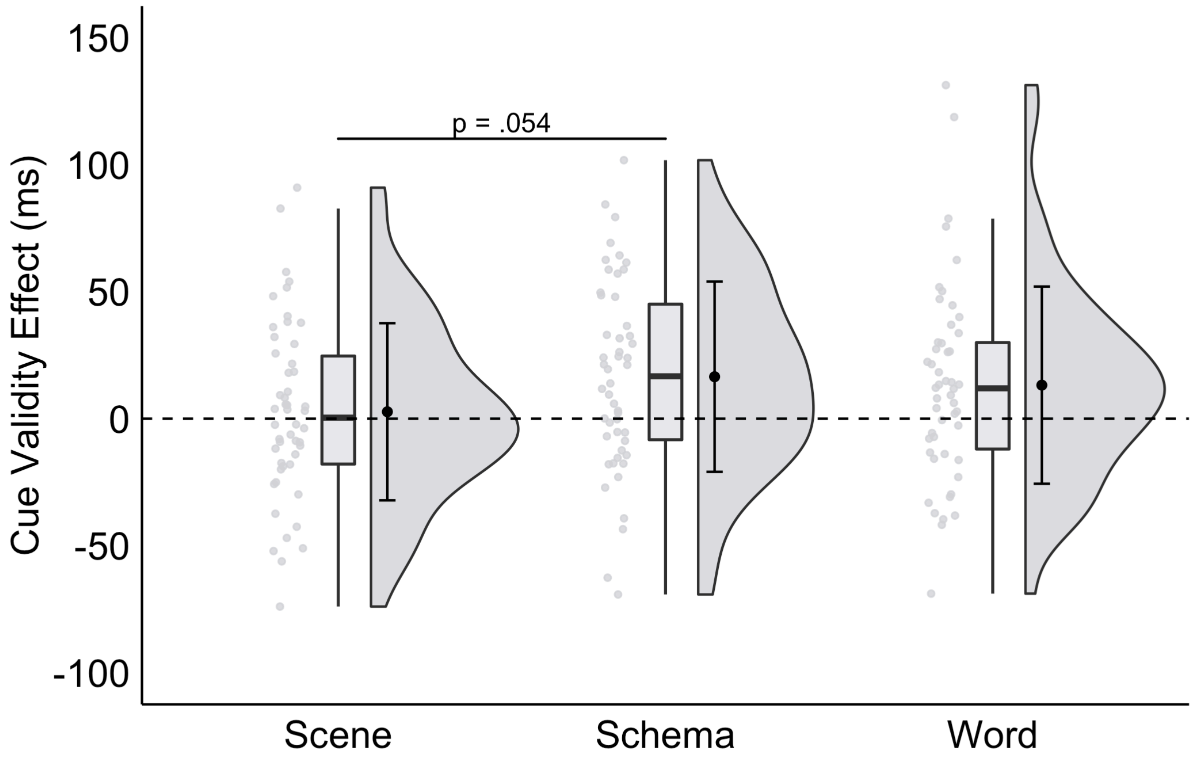

Figure S7. **No significant interaction between trial type and cue format for participants recruited from Prolific in Experiment 1**, plotted as the cue validity effect as a function of cue format.
Note: critical α = .05.

Exploratory Results. To explore differences in how attention was allocated with cues that provided spatial information of the upcoming target (i.e., left or right spatial directions)

compared to cues that did not provide any spatial information of the upcoming target (i.e., ahead spatial direction; neutral trials), RTs were submitted to a 2 (target location: left, right) x 3 (cue direction: ahead, left, right) x 3 (cue format: scene, schema, word) within-subjects repeated measures ANOVA. No main effects or interactions were significant (*F*s < 0.6, *p*s > .56), except for the interaction between target location and cue direction, $F(2,98) = 10.53$, $p < .001$, $\eta_p^2 = 0.18$, $BF_{10} = 404.57$, which was driven by differences in RTs on trials with either a left or right cue as a function of target location (See the upper left plot in Figure S8). In particular, for trials with a left cue, RTs were significantly faster when the target appeared on the left ($M = 388.49$ ms, $SEM = 4.35$ ms) than the right ($M = 402.44$ ms, $SEM = 5.58$ ms), $t(149) = 3.51$, $p < .001$, $d = 0.22$, $BF_{10} = 30.08$. In contrast, for trials with a right cue, RTs were significantly faster when the target appeared on the right ($M = 390.76$ ms, $SEM = 4.28$ ms) than the left ($M = 399.17$ ms, $SEM = 5.28$ ms), $t(149) = 2.14$, $p = .034$, $d = 0.14$, $BF_{10} = 0.83$. Critically, for trials with an ahead cue, RTs were statistically equivalent when the target appeared on the left ($M = 397.60$ ms, $SEM = 4.62$ ms) or the right ($M = 396.78$ ms, $SEM = 4.41$ ms), $t(149) = 0.30$, $p = .764$, $d = 0.01$, $BF_{10} = 0.10$. The interaction between target location, cue direction, and cue format was not significant, $F(4,196) = 1.78$, $p = .134$, $\eta_p^2 = 0.04$, $BF_{10} = 0.17$ (See the upper right, lower left, and lower right plot in Figure S8 and Table S4).

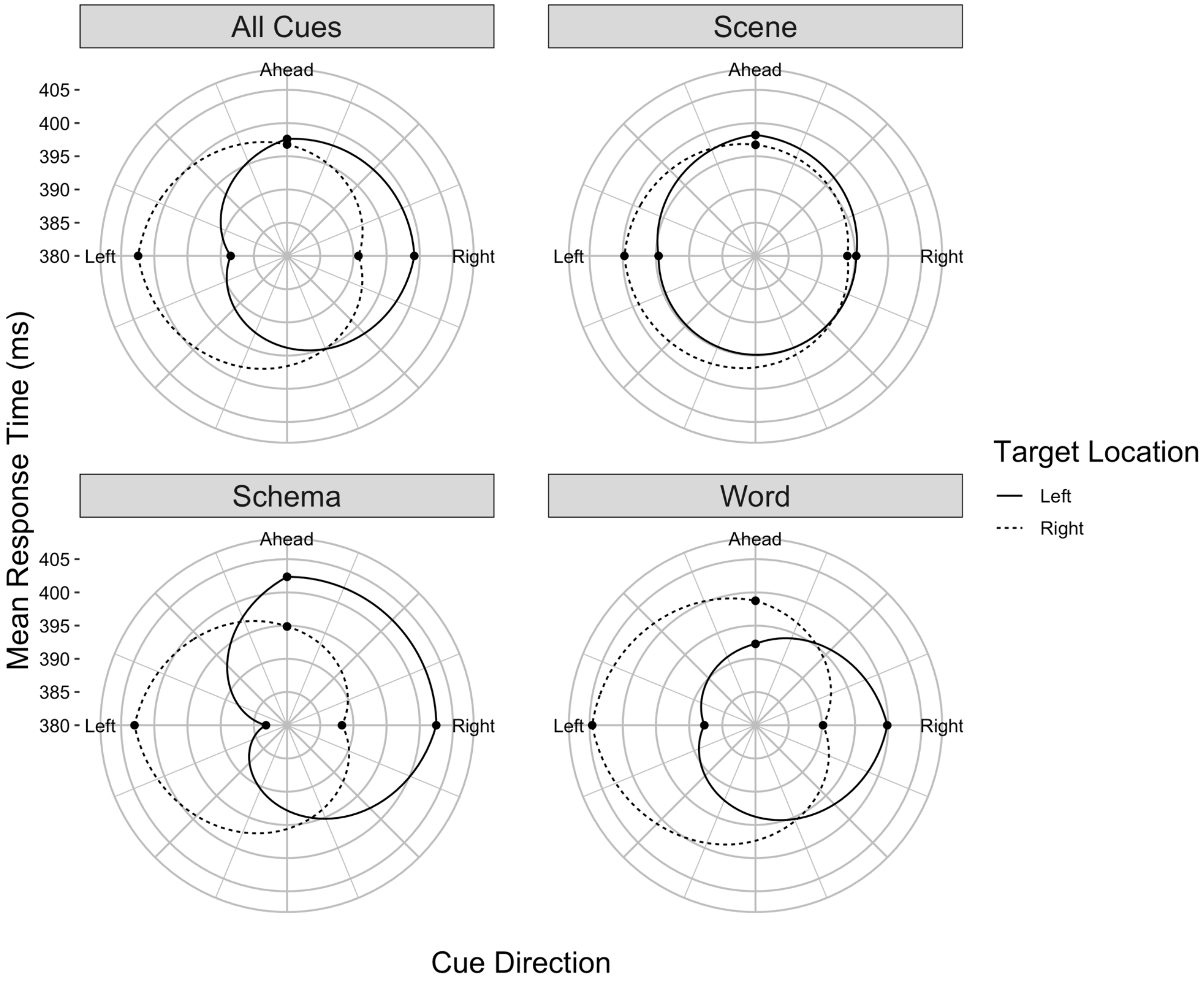

Figure S8. **Exploratory results for participants recruited from Prolific in Experiment 1.**

Table S4. *RTs as a function of cue format, cue direction, and target location for participants recruited from Prolific in Experiment 1.*

Cue Format	Cue Direction	*M* (*SEM*) RT for Left Targets (ms)	*M* (*SEM*) RT for Right Targets (ms)	*t*	*p*	*d*	JZS Bayes factor (BF_{10})
	Ahead	398.20 (8.14)	396.73 (6.82)	0.35	.726	0.03	0.16
Scene	Left	394.60 (7.75)	399.75 (8.89)	0.73	.468	0.09	0.20
	Right	395.18 (9.77)	393.84 (7.44)	0.19	.848	0.02	0.16
	Ahead	402.35 (8.11)	394.87 (8.50)	1.38	.173	0.13	0.38
Schema	**Left**	**383.17 (7.27)**	**402.98 (9.11)**	**3.08**	**.003**	**0.33**	**9.54**
	**Right**	**402.48 (8.32)**	**388.27 (7.59)**	**2.06**	**.045**	**0.25**	**1.06**
	Ahead	392.25 (7.88)	398.74 (7.66)	1.46	.150	0.12	0.42
Word	**Left**	**387.20 (7.63)**	**404.60 (11.04)**	**2.37**	**.022**	**0.23**	**1.93**
	Right	399.86 (9.45)	390.18 (7.33)	1.48	.146	0.16	0.42

Note: Significant differences between left and right targets are bolded (critical $\alpha = .05$).

Experiment 1 – Exploratory Results

Cues depicting left and right spatial directions preferentially directed participants' space-based attention to a location where the upcoming target was likely to appear (with 70% validity), whereas cues depicting the ahead spatial direction did not provide any relevant information about the location of the upcoming target (i.e., there was equal probability of the target appearing on the left or right side of the screen). Thus, to explore differences in how attention was allocated with cues that provided spatial information of the upcoming target (i.e., left or right spatial directions) compared to cues that did not provide any spatial information of the upcoming target (i.e., ahead spatial direction; neutral trials), RTs were submitted to a 2 (target location: left, right) x 3 (cue direction: ahead, left, right) x 3 (cue format: scene, schema, word) within-subjects repeated measures ANOVA. There were no significant main effects of target location, cue direction, or cue format, or significant interactions between target location and cue format or between cue direction and cue format, *F*s < 1.0, *p*s > .45. However, there was a significant interaction between target location and cue direction, $F(2,198) = 14.50$, $p < .001$, $\eta_p^2 = 0.13$, $BF_{10} = 3{,}013{,}560$, that was driven by differences in RTs on trials with either a left or right cue as a function of target location (see the upper left plot in Figure S9). In particular, for trials with a left cue, RTs were significantly faster when the target appeared on the left ($M = 387.17$ ms, $SEM = 3.02$ ms) than the right ($M = 399.70$ ms, $SEM = 3.86$ ms), $t(299) = 4.34$, $p < .001$, $d = 0.20$, $BF_{10} = 547.64$. In contrast, for trials with a right cue, RTs were significantly faster when the target appeared on the right ($M = 389.38$ ms, $SEM = 3.17$ ms) than the left ($M = 401.42$ ms, $SEM = 4.29$ ms), $t(299) = 3.70$, $p < .001$, $d = 0.18$, $BF_{10} = 48.35$. That is, space-based attention was preferentially allocated to the left following a left cue, resulting in faster detection of the target on the left than the right; the reverse was true for targets on the right following a right cue.

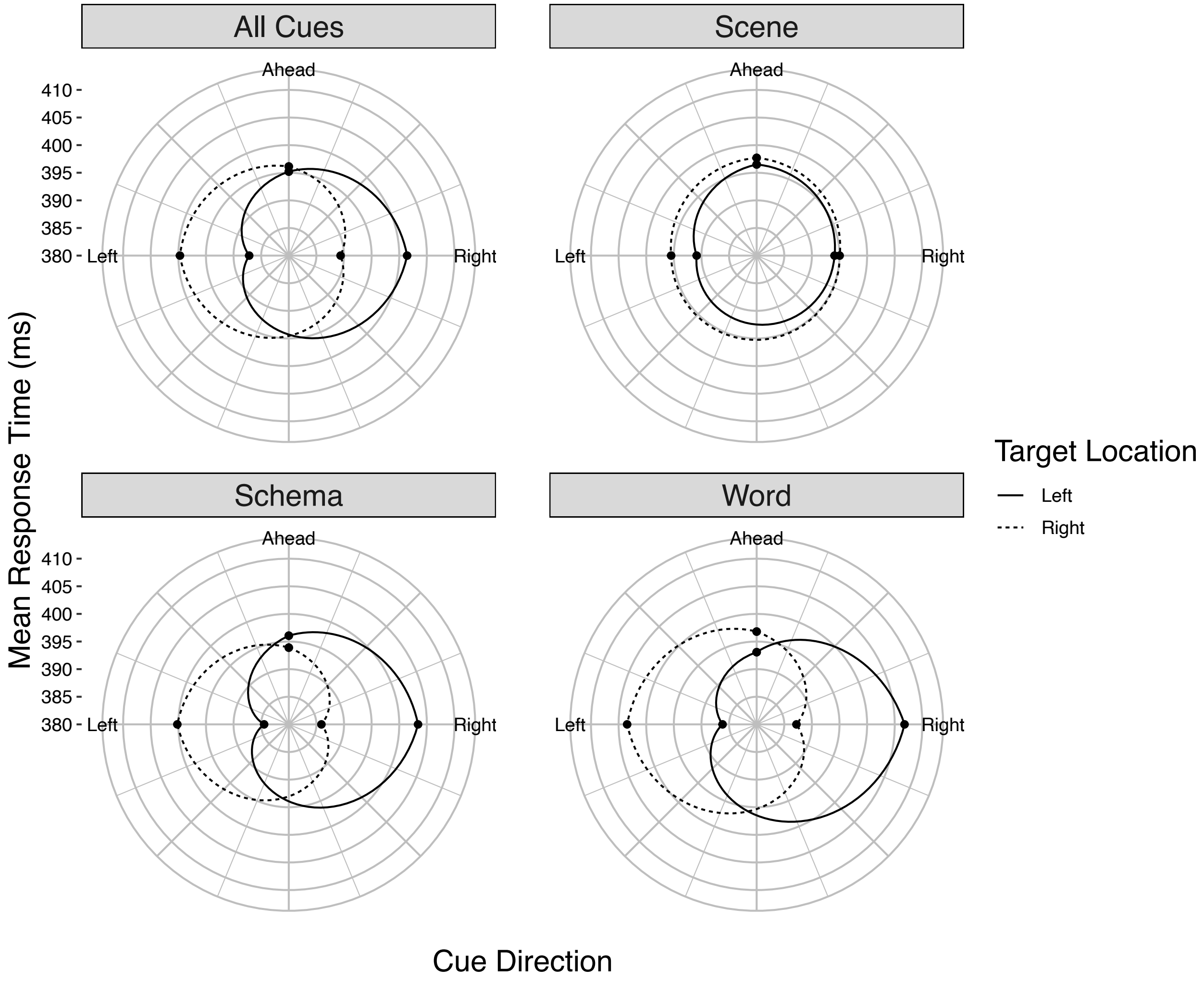

Figure S9. **Exploratory results from Experiment 1.**

Critically, for trials with an ahead cue, RTs were statistically equivalent when the target appeared on the left (*M* = 395.21 ms, *SEM* = 3.39 ms) or the right (*M* = 396.13 ms, *SEM* = 3.16 ms), $t(299) = 0.43$, $p = .667$, $d = 0.02$, $BF_{10} = 0.07$. Thus, as expected, ahead cues did not provide an attentional benefit for detecting the target.

The interaction between target location, cue direction, and cue format was also significant, $F(4,396) = 4.21$, $p = .002$, $\eta_p^2 = 0.04$, $BF_{10} = 3.23$, indicating that the interaction between target location and cue direction was further modulated by the format of the cue (see

Table S5). Specifically, for left schema and word cues (see the lower left and lower right plots in Figure S9, respectively), RTs were significantly faster when the target appeared on the left than the right. Conversely, for right schema and word cues, RTs were significantly faster when the target appeared on the right than the left. For ahead schema and word cues, there were no differences between RTs when the target appeared on the left or the right. However, for scene cues (see the upper right plot in Figure S9), there were no differences between RTs when the target appeared on the left or the right regardless of the spatial direction indicated by the cue.

Table S5. *RTs as a function of cue format, cue duration, and target location in Experiment 1.*

Cue Format	Cue Direction	*M* (*SEM*) RT for Left Targets (ms)	*M* (*SEM*) RT for Right Targets (ms)	*t*	*p*	*D*	JZS Bayes factor (BF_{10})
	Ahead	396.50 (5.84)	397.69 (5.59)	0.32	.750	0.02	0.12
Scene	Left	390.86 (5.04)	395.48 (6.39)	0.89	.374	0.08	0.16
	Right	394.10 (7.26)	395.03 (5.68)	0.17	.863	0.01	0.11
	Ahead	396.06 (5.63)	393.89 (5.65)	0.60	.551	0.04	0.13
Schema	**Left**	**384.47 (5.25)**	**400.18 (6.00)**	**3.56**	**< .001**	**0.28**	**36.47**
	**Right**	**403.40 (7.32)**	**385.91 (5.39)**	**3.06**	**.003**	**0.26**	**8.54**
	Ahead	393.07 (6.19)	396.80 (5.22)	0.99	.323	0.06	0.18
Word	**Left**	**386.18 (5.40)**	**403.45 (7.58)**	**3.24**	**.002**	**0.25**	**14.47**
	**Right**	**406.77 (7.70)**	**387.20 (5.42)**	**3.44**	**< .001**	**0.28**	**25.70**

Note: Significant differences between left and right targets are bolded (critical $\alpha = .035$).

Experiment 2 – False Alarm and Miss Rates

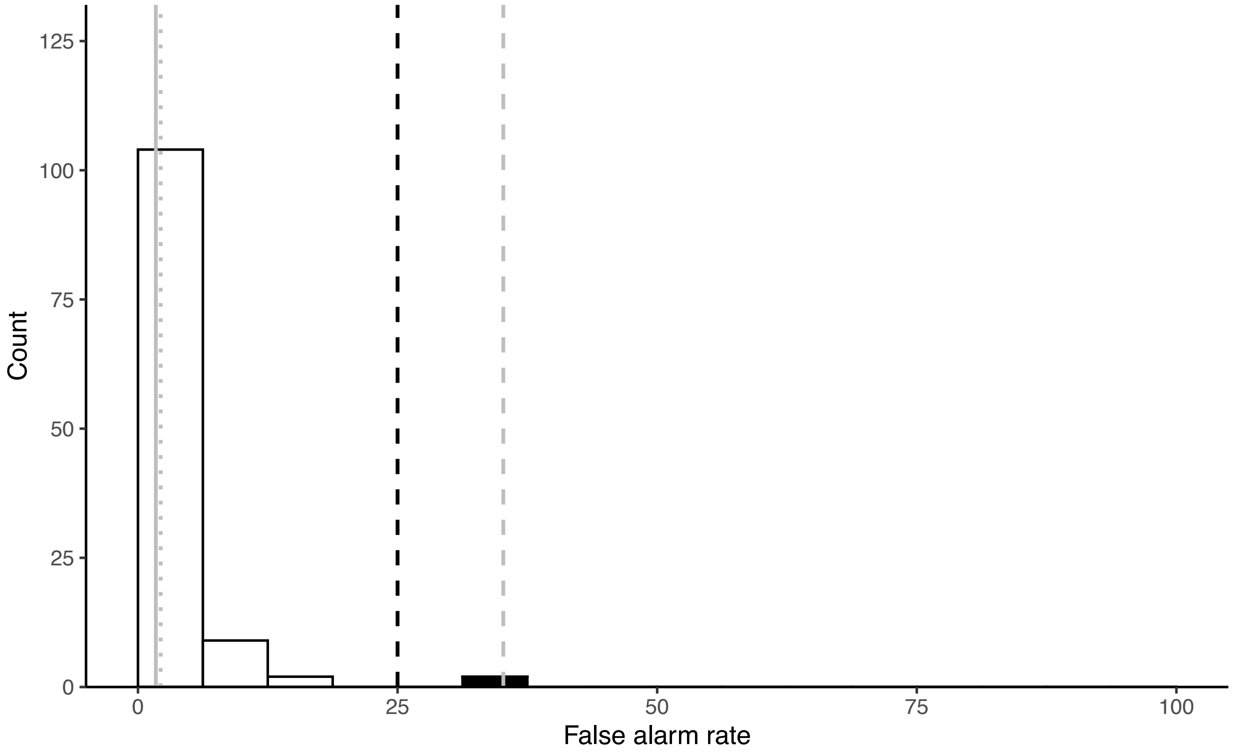

Figure S10. **Histogram of participant false alarm rates from Experiment 2.** The dashed black line at 25% marks the cutoff – participants with a false alarm rate greater than 25% were excluded. Two participants had a false alarm rate greater than 25% (black filled bars). The dotted gray line indicates the average false alarm rate for all 117 participants (2.18%). The dashed gray line indicates the average false alarm rate for the two excluded participants (35.18%). The solid gray line indicates the average false alarm rate for the 101 included participants (1.72%). A Welch independent samples *t*-test revealed a significantly larger false alarm rate for the excluded participants than the 101 included participants, $t(1.06) = 17.80$, $p = .03$, $d = 10.30$.

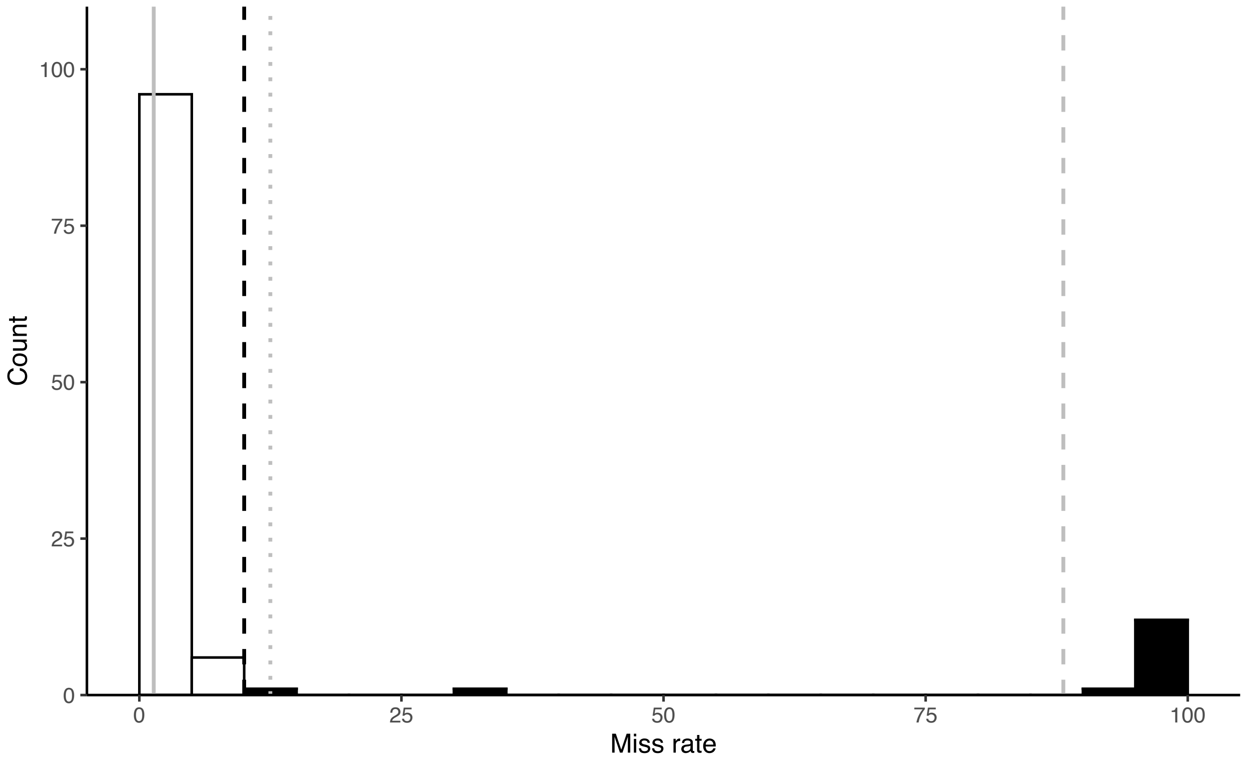

***Figure S11*. Histogram of participant miss rates from Experiment 2.** The dashed black line at 10% marks the cutoff – participants with a miss rate greater than 10% were excluded. Fifteen participants had a miss rate greater than 10% (black filled bars). The dotted gray line indicates the average miss rate for all 117 participants (12.50 %). The dashed gray line indicates the average miss rate for the 15 excluded participants (88.13%). The solid gray line indicates the average miss rate for the 101 included participants (1.64%). A Welch independent samples t-test revealed a significantly larger miss rate for the excluded participants than the 101 included participants, $t(14.03) = 12.36$, $p < .001$, $d = 8.92$.

Experiment 2 – Exploratory Results

To explore differences in how attention was allocated with cues that provided spatial information of the upcoming target (i.e., left or right spatial directions) compared to cues that did not provide any spatial information of the upcoming target (i.e., ahead spatial direction; neutral trials), RTs were submitted to a 2 (target location: left, right) x 3 (cue direction: ahead, left, right) x 3 (cue format: scene, schema, word) within-subjects repeated measures ANOVA. There were no significant main effects of target location, cue direction, or cue format, or significant interactions between target location and cue format or between cue direction and cue format, *F*s < 2.1, *p*s $> .13$. Consistent with Experiment 1, the interaction between target location and cue direction was significant, $F(2,200) = 23.86$, $p < .001$, $\eta_p^2 = 0.19$, $BF_{10} = 24{,}675{,}284$, which was driven by differences in RTs on trials with either a left or right cue as a function of target location (See the upper left plot in Figure S12). In particular, for trials with a left cue, RTs were significantly faster when the target appeared on the left ($M = 397.77$ ms, $SEM = 5.28$ ms) than the right ($M = 412.71$ ms, $SEM = 6.04$ms), $t(302) = 4.86$, $p < .001$, $d = 0.22$, $BF_{10} = 1{,}466.01$. In contrast, for trials with a right cue, RTs were significantly faster when the target appeared on the right ($M = 398.86$ ms, $SEM = 5.60$ ms) than the left ($M = 409.67$ ms, $SEM = 5.60$ ms), $t(302) = 3.86$, $p < .001$, $d = 0.17$, $BF_{10} = 31.36$. Critically, for trials with an ahead cue, RTs were statistically equivalent when the target appeared on the left ($M = 406.33$ ms, $SEM = 5.44$ ms) or the right ($M = 407.43$ ms, $SEM = 5.12$ ms), $t(302) = 0.52$, $p = .418$, $d = 0.61$, $BF_{10} = 0.12$.

The interaction between target location, cue direction, and cue format was also significant, $F(4,400) = 4.30$, $p = .002$, $\eta_p^2 = 0.04$, $BF_{10} = 2.85$, indicating that the interaction between target location and cue direction was further modulated by the format of the cue (See Figure S12 and Table S6). Specifically, for right schema cues (See the lower left plot in Figure

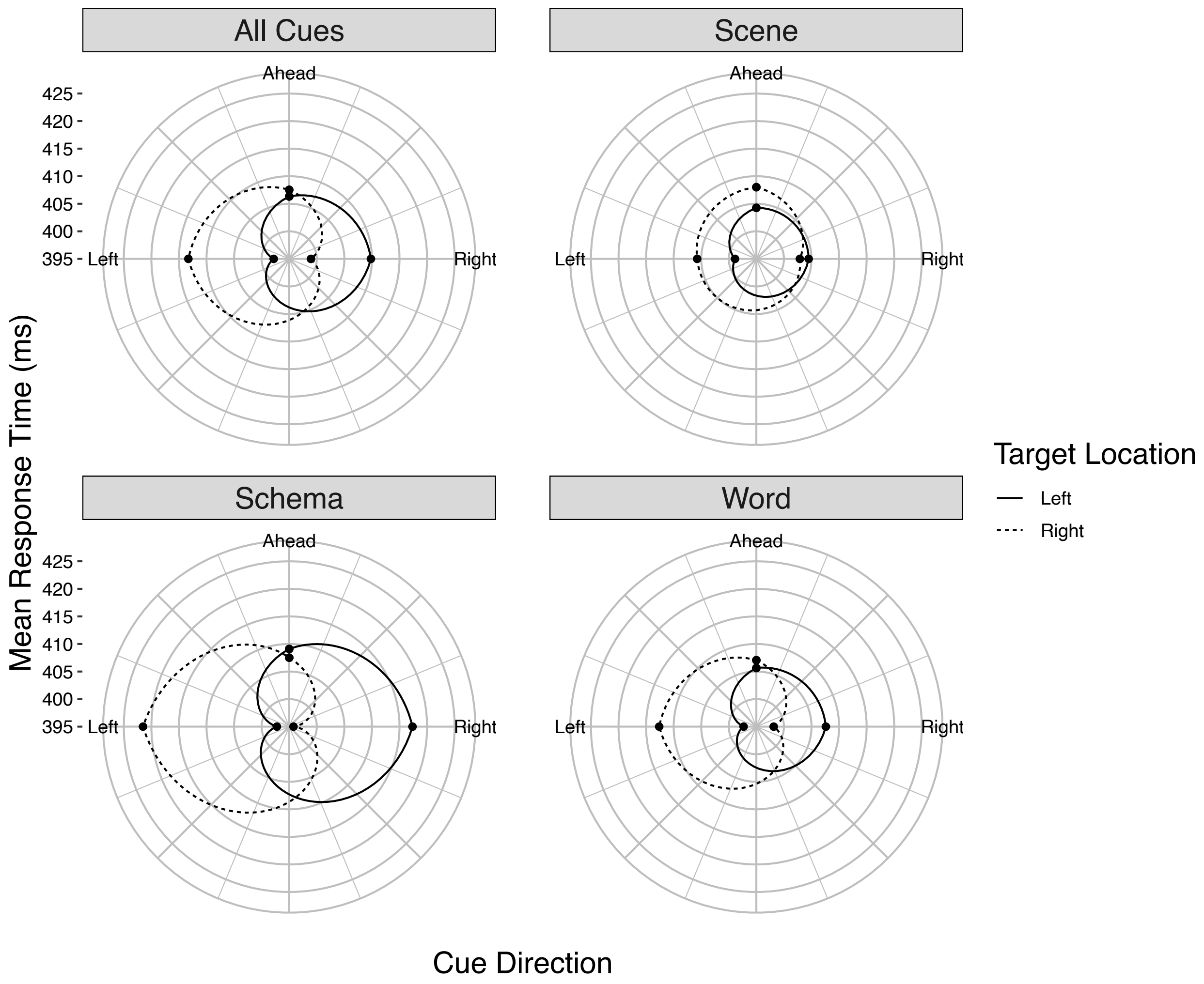

Figure S12. **Exploratory results from Experiment 2.**

S12), RTs were significantly faster when the target appeared on the right than the left. This effect was marginally significant for word cues (See the lower right plot in Figure S12). Conversely, for left schema and word cues, RTs were significantly faster when the target appeared on the left than the right. For ahead schema and word cues, there were no differences between RTs when the target appeared on the left or the right. However, for scene cues (See the upper right plot in Figure S12), there were no differences between RTs when the target appeared on the left or the right regardless of the spatial direction indicated by the cue.

Table S6. *RTs as a function of cue format, cue direction, and target location in Experiment 2.*

Cue Format	Cue Direction	*M* (*SEM*) RT for Left Targets (ms)	*M* (*SEM*) RT for Right Targets (ms)	*t*	*p*	*d*	JZS Bayes factor (BF_{10})
Scene	Ahead	404.26 (5.49)	408.01 (5.83)	0.85	.399	0.07	0.16
	Left	398.87 (5.08)	405.74 (6.75)	1.33	.185	0.11	0.26
	Right	404.49 (6.15)	402.89 (5.76)	0.34	.732	0.03	0.12
Schema	Ahead	409.09 (6.22)	407.51 (5.24)	0.40	.691	0.03	0.12
	**Left**	**397.24 (5.83)**	**421.51 (8.57)**	**3.91**	**< .001**	**0.31**	**113.11**
	**Right**	**417.36 (6.91)**	**395.77 (6.09)**	**4.30**	**< .001**	**0.33**	**425.25**
Word	Ahead	405.64 (5.94)	407.06 (5.52)	0.40	.693	0.02	0.12
	**Left**	**397.31 (5.79)**	**412.62 (7.40)**	**3.04**	**.003**	**0.22**	**8.22**
	Right	*407.61 (6.50)*	*398.14 (5.60)*	*1.96*	*.052*	*0.15*	*0.69*

Note: Significant differences between left and right targets are bolded, and marginal differences are italicized (critical $\alpha = .035$).

Experiment 3 – False Alarm and Miss Rates

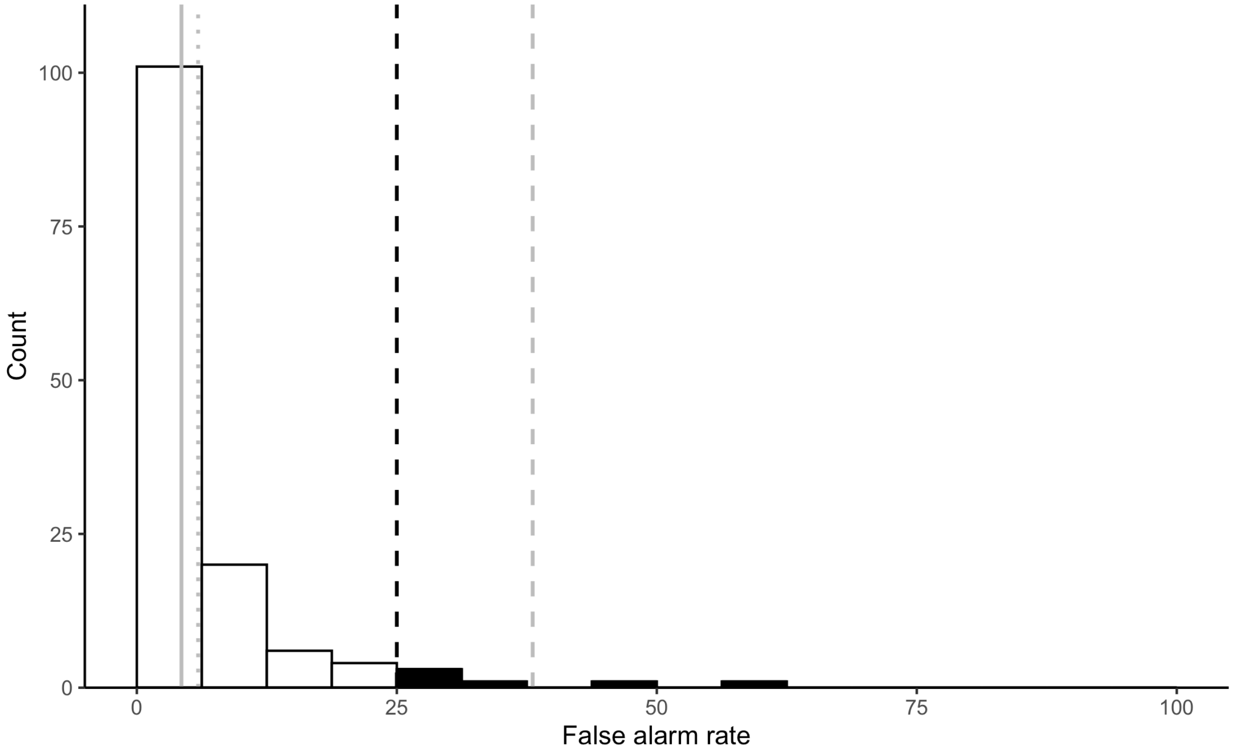

***Figure S13*. Histogram of participant false alarm rates from Experiment 3.** The dashed black line at 25% marks the cutoff – participants with a false alarm rate greater than 25% were excluded. Six participants had a false alarm rate greater than 25% (black filled bars). The dotted gray line indicates the average false alarm rate for all 137 participants (5.89%). The dashed gray line indicates the average false alarm rate for the six excluded participants (38.07%). The solid gray line indicates the average false alarm rate for the 101 included participants (4.29%). A Welch independent samples *t*-test revealed a significantly larger false alarm rate for the excluded participants than the 101 included participants, $t(38.19) = 2.51$, $p = .017$, $d = 0.72$.

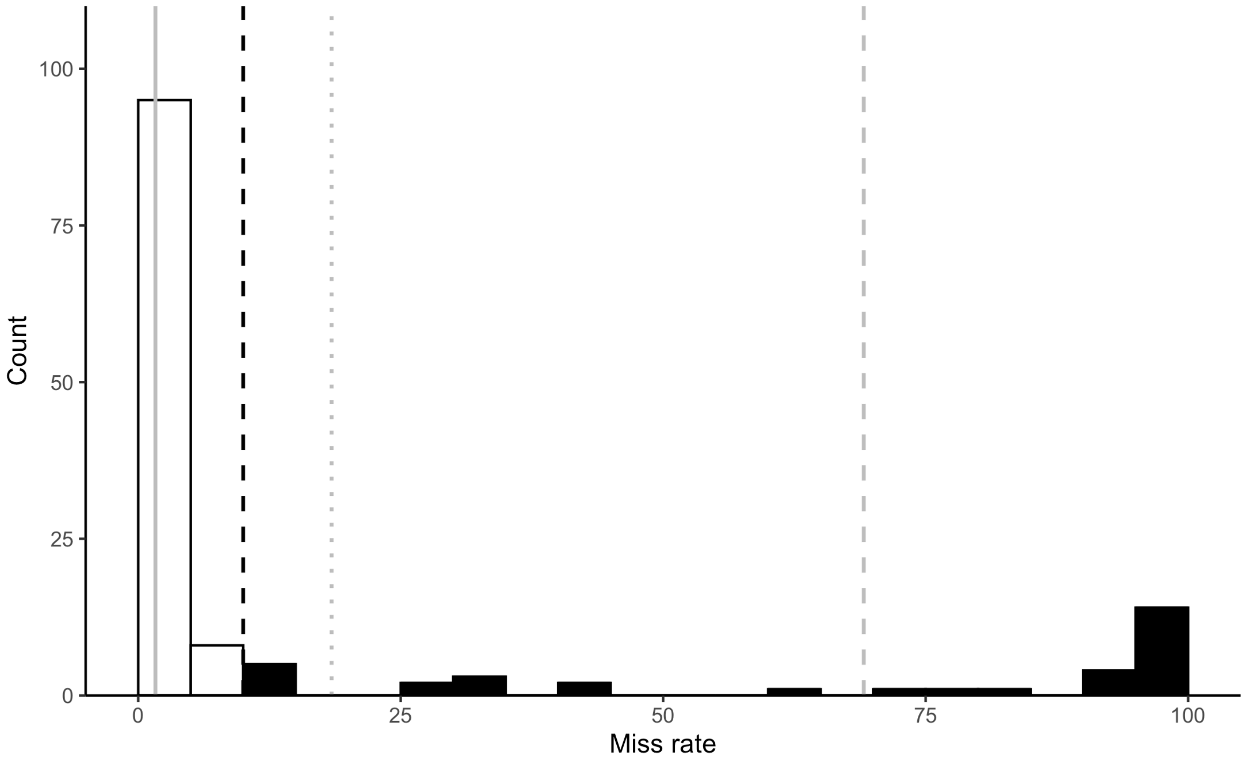

***Figure S14*. Histogram of participant miss rates from Experiment 3.** The dashed black line at 10% marks the cutoff – participants with a miss rate greater than 10% were excluded. Thirty-four participants had a miss rate greater than 10% (black filled bars). The dotted gray line indicates the average miss rate for all 137 participants (18.42%). The dashed gray line indicates the average miss rate for the 34 excluded participants (69.10%). The solid gray line indicates the average miss rate for the 101 included participants (1.64%). A Welch independent samples *t*-test revealed a significantly larger miss rate for the excluded participants than the 101 included participants, $t(35.07) = 10.35$, $p < .001$, $d = 3.38$.

Experiment 3 – Additional Cue Duration Results

The interaction between cue format and cue duration was not significant, $F(4,400) = 0.45$, $p = .770$, $\eta_p^2 = 0.01$, $BF_{10} = 0.002$ (all pairwise comparisons, *t*s < 1.3, *p*s > .20). The interaction between trial type and cue duration was also not significant, $F(2,200) = 0.29$, $p = .752$, $\eta_p^2 = 0.003$, $BF_{10} = 0.02$. However, pairwise comparisons revealed that RTs on valid trials ($M_{600\text{ ms}} = 375.37$ ms, $SEM_{600\text{ ms}} = 2.67$ ms; $M_{1200\text{ ms}} = 379.27$ ms, $SEM_{1200\text{ ms}} = 2.73$ ms; $M_{2400\text{ ms}} = 386.42$ ms, $SEM_{2400\text{ ms}} = 2.67$ ms) were significantly faster than RTs on invalid trials ($M_{600\text{ ms}} = 380.67$ ms, $SEM_{600\text{ ms}} = 2.98$ ms; $M_{1200\text{ ms}} = 386.92$ ms, $SEM_{1200\text{ ms}} = 3.29$ ms; $M_{2400\text{ ms}} = 393.29$ ms, $SEM_{2400\text{ ms}} = 3.23$ ms) for all three cue durations (*t*s > 2.4, *p*s < .018, *d*s > 0.12, BF_{10}s > 1.10. This finding reveals that cue validity effects were present for all three cue durations. Furthermore, there were no significant differences in the cue validity effect between the cue durations ($M_{600\text{ ms}} = 5.31$ ms, $SEM_{600\text{ ms}} = 2.19$ ms; $M_{1200\text{ ms}} = 7.65$ ms, $SEM_{1200\text{ ms}} = 2.27$ ms; $M_{2400\text{ ms}} = 6.87$ ms, $SEM_{2400\text{ ms}} = 2.37$ ms), all *t*s < 0.8, all *p*s > .45 (see Figure S15). In other words, cue duration did not impact the cue validity effect.

The interaction between trial type, cue format, and cue duration was not significant, $F(4,400) = 1.11$, $p = .350$, $\eta_p^2 = 0.01$, $BF_{10} = 0.02$. (See Table S7 for pairwise comparisons between valid and invalid trials as a function of cue format and cue duration.) Notably, valid trials were significantly faster than invalid trials for schema cues presented at all three cue durations, as well as for word cues presented at 1200 ms. When the cue validity effect was calculated for each cue format and cue duration, no significant pairwise comparisons were found, all *t*s < 1.4, all *p*s > .18 (see Figure S16). This finding indicates that cue duration did not modulate preferential allocation of space-based attention for scenes, schemas, or words.

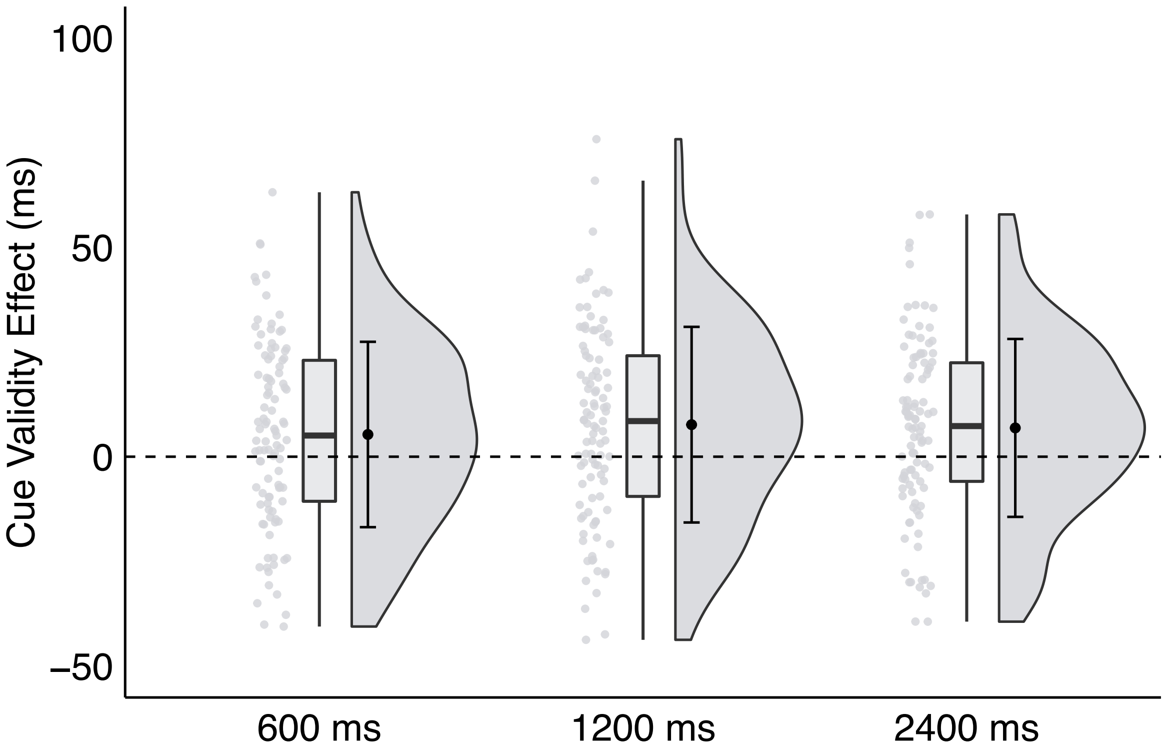

Figure S15. **No significant interaction between trial type and cue duration in Experiment 3**, plotted as the cue validity effect as a function of cue duration.

Table S7. *RTs as a function of cue format, cue duration, and trial type in Experiment 3.*

Cue Format	Cue Duration	*M* (*SEM*) RT for Valid Trials (ms)	*M* (*SEM*) RT for Invalid Trials (ms)	*M* (*SEM*) Cue Validity Effect (ms)	*t*	*p*	*D*	JZS Bayes factor (BF_{10})
	600 ms	378.67 (4.52)	375.17 (5.00)	-3.50 (3.56)	0.98	.328	0.07	0.18
Scene	1200 ms	382.68 (4.90)	380.11 (5.22)	-2.57 (3.58)	0.72	.474	0.05	0.14
	2400 ms	388.96 (4.43)	391.93 (5.27)	2.98 (3.91)	0.76	.449	0.06	0.15
	600 ms	**369.89 (4.44)**	**387.48 (5.29)**	**17.59 (3.80)**	**4.63**	**< .001**	**0.35**	**1429.06**
Schema	1200 ms	**374.46 (4.64)**	**390.77 (5.71)**	**16.31 (3.99)**	**4.08**	**< .001**	**0.30**	**203.29**
	2400 ms	**383.53 (4.85)**	**394.61 (5.50)**	**11.08 (4.14)**	**2.68**	**.009**	**0.21**	**3.21**
	600 ms	377.54 (4.89)	379.37 (5.18)	1.83 (3.71)	0.49	.624	0.03	0.12
Word	1200 ms	**380.67 (4.66)**	**389.87 (6.12)**	**9.21 (4.02)**	**2.29**	**.024**	**0.16**	**1.33**
	2400 ms	386.76 (4.62)	393.32 (6.02)	6.56 (4.27)	1.54	.128	0.12	0.34

Note: Significant differences between valid and invalid trials (cue validity effect) are bolded (critical $\alpha = .035$).

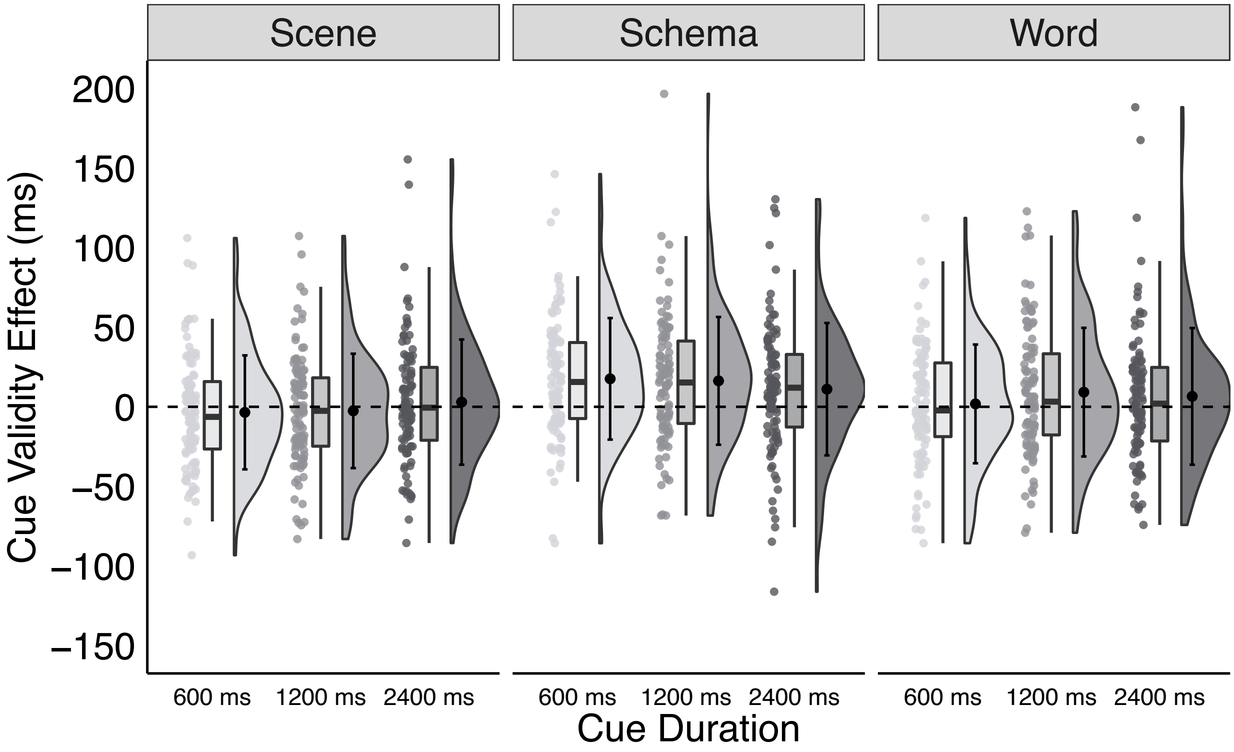

Figure S16. **No significant interaction between trial type, cue format, and cue duration in Experiment 3**, plotted as the cue validity effect as a function of cue duration for each cue format.

Experiment 3 – Exploratory Results

To explore differences in how attention was allocated with cues that provided spatial information of the upcoming target (i.e., left or right spatial directions) compared to cues that did not provide any spatial information of the upcoming target (i.e., ahead spatial direction; neutral trials), RTs were submitted to a 2 (target location: left, right) x 3 (cue direction: ahead, left, right) x 3 (cue format: scene, schema, word) within-subjects repeated measures ANOVA. There were no significant main effects of target location, cue direction, or cue format, or significant interactions between target location and cue format or between cue direction and cue format, *F*s < 2.7, *p*s $> .07$. However, there was a significant interaction between target location and cue direction, $F(2,200) = 17.47$, $p < .001$, $\eta_p^2 = 0.15$, $BF_{10} = 41,780.23$, that was driven by differences in RTs on trials with either a left or right cue as a function of target location (See the upper left plot in Figure S17). In particular, for trials with a left cue, RTs were significantly faster when the target appeared on the left ($M = 380.02$ ms, $SEM = 2.60$ ms) than the right ($M = 389.12$ ms, $SEM = 2.96$ ms), $t(302) = 4.26$, $p < .001$, $d = 0.19$, $BF_{10} = 390.75$. In contrast, for trials with a right cue, RTs were significantly faster when the target appeared on the right ($M = 380.48$ ms, $SEM = 2.55$ ms) than the left ($M = 385.43$ ms, $SEM = 2.78$ ms), $t(302) = 2.61$, $p = .009$, $d = 0.11$, $BF_{10} = 1.81$. Critically, for trials with an ahead cue, RTs were statistically equivalent when the target appeared on the left ($M = 385.23$ ms, $SEM = 2.75$ ms) or the right ($M = 386.33$ ms, $SEM = 2.66$ ms), $t(302) = 0.76$, $p = .449$, $d = 0.02$, $BF_{10} = 0.09$.

The interaction between target location, cue direction, and cue format was also significant, $F(4,400) = 7.51$, $p < .001$, $\eta_p^2 = 0.07$, $BF_{10} = 1,478.00$, indicating that the interaction between target location and cue direction was further modulated by the format of the cue (See Figure S17 and Table S8). Specifically, for left schema and word cues (See the lower left and

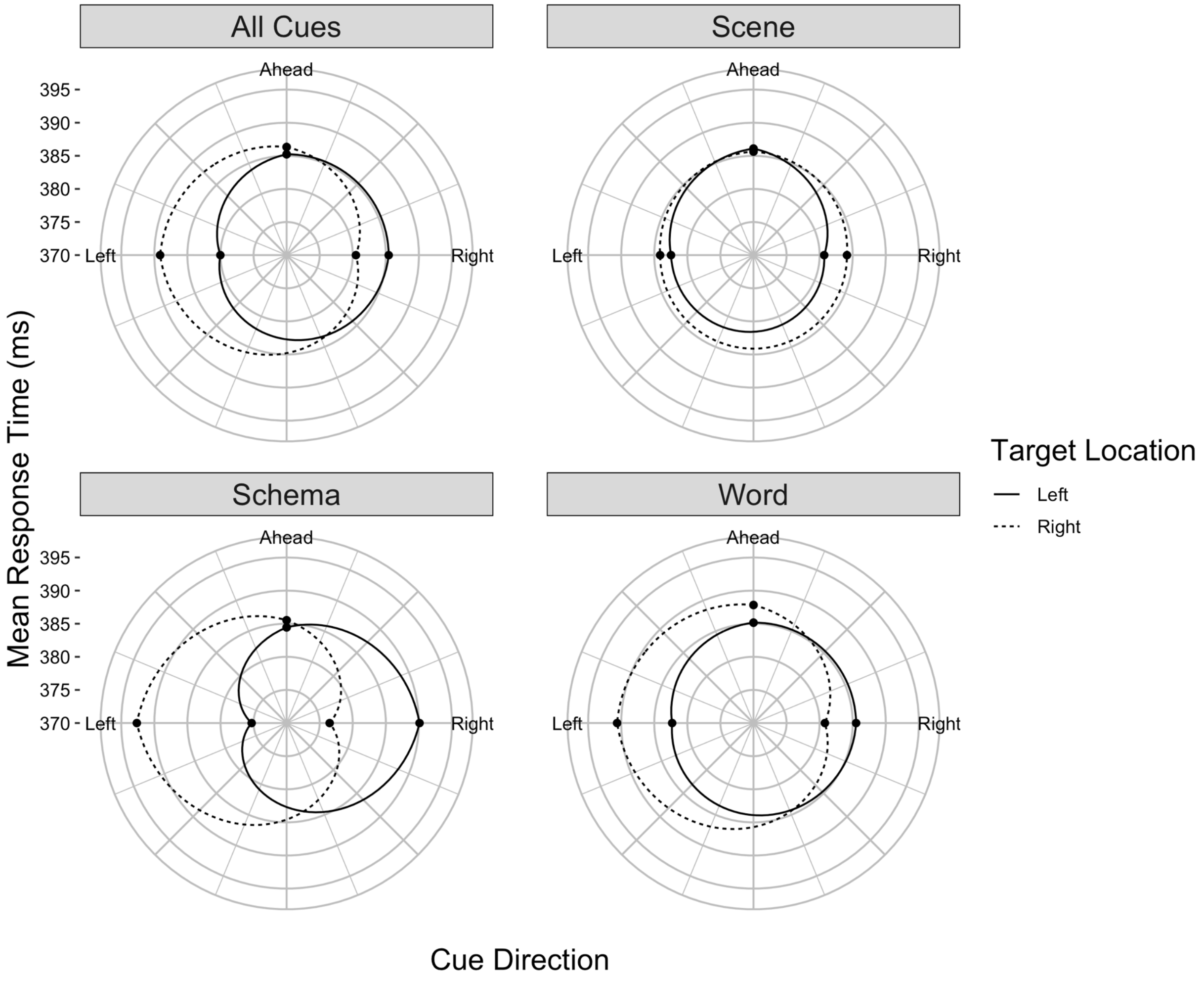

Figure S17. **Exploratory results from Experiment 3.**

lower right plots in Figure S17, respectively), RTs were significantly faster when the target appeared on the left than the right. Conversely, for right schema cues, RTs were significantly faster when the target appeared on the right than the left. A similar pattern emerged for right word cues, but the difference did not reach significance. For ahead schema and word cues, there were no differences between RTs when the target appeared on the left or the right. However, for scene cues (See the upper right plot in Figure S17), there were no differences between RTs when the target appeared on the left or the right regardless of the spatial direction indicated by the cue.

Table S8. *RTs as a function of cue format, cue direction, and target location from Experiment 3.*

Cue Format	Cue Direction	*M* (*SEM*) RT for Left Targets (ms)	*M* (*SEM*) RT for Right Targets (ms)	*t*	*p*	*d*	JZS Bayes factor (BF_{10})
Scene	Ahead	386.09 (4.71)	385.63 (4.41)	0.21	.833	0.01	0.11
	Left	382.46 (4.30)	384.11 (4.59)	0.46	.646	0.04	0.12
	Right	380.69 (4.76)	384.13 (4.43)	1.04	.303	0.07	0.19
Schema	Ahead	384.44 (4.65)	385.52 (4.83)	0.37	.713	0.02	0.12
	**Left**	**375.29 (4.43)**	**392.64 (5.34)**	**4.33**	**< .001**	**0.35**	**485.28**
	**Right**	**390.09 (4.66)**	**376.51 (4.43)**	**4.37**	**< .001**	**0.30**	**548.43**
Word	Ahead	385.16 (4.95)	387.82 (4.63)	1.11	.268	0.05	0.20
	**Left**	**382.30 (4.74)**	**390.62 (5.44)**	**2.46**	**.016**	**0.16**	**1.93**
	Right	385.51 (5.02)	380.80 (4.38)	1.46	.146	0.10	0.31

Note: Significant differences between left and right targets are bolded (critical $\alpha = .035$).

Experiment 4 – False Alarm and Miss Rates

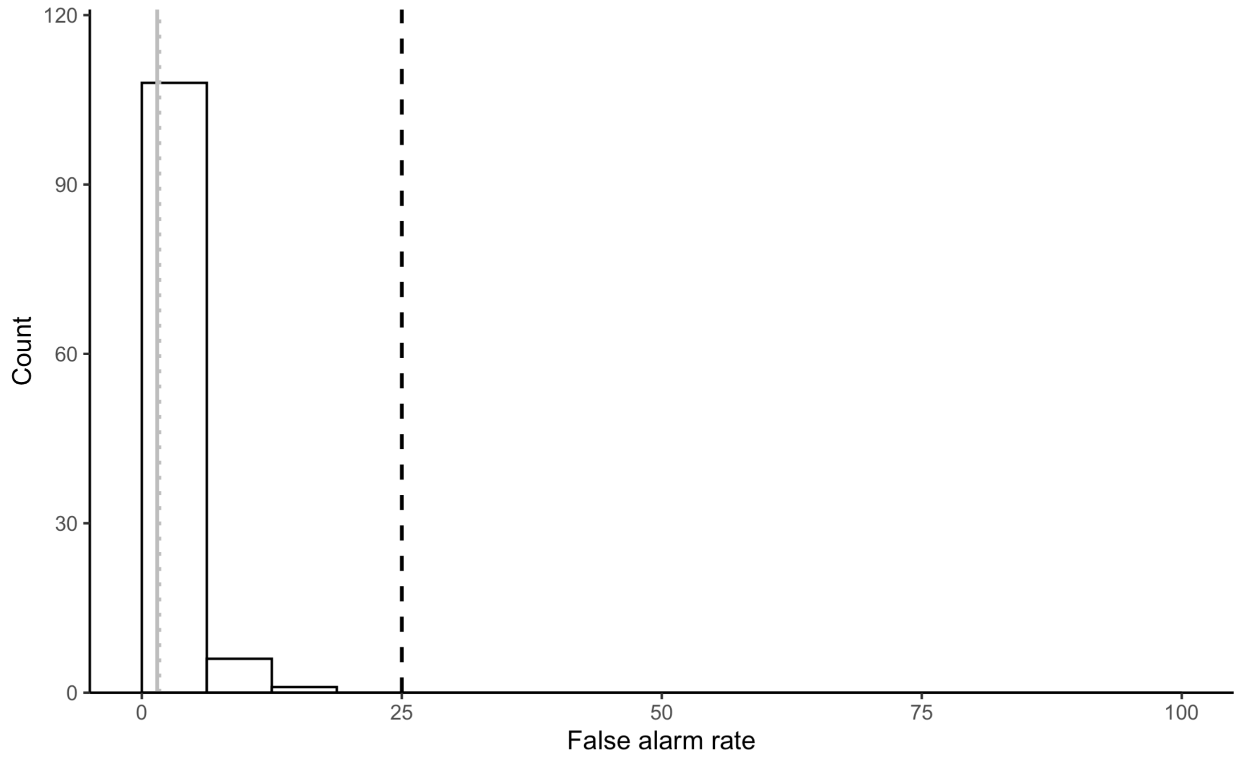

***Figure S18.* Histogram of participant false alarm rates from Experiment 4.** The dashed black line at 25% marks the cutoff – participants with a false alarm rate greater than 25% were excluded. No participants had a false alarm rate greater than 25%. The dotted gray line indicates the average false alarm rate for all 115 participants (1.68%). The solid gray line indicates the average false alarm rate for the 101 included participants (1.48%).

Note. Fourteen participants were excluded due to high miss rates.

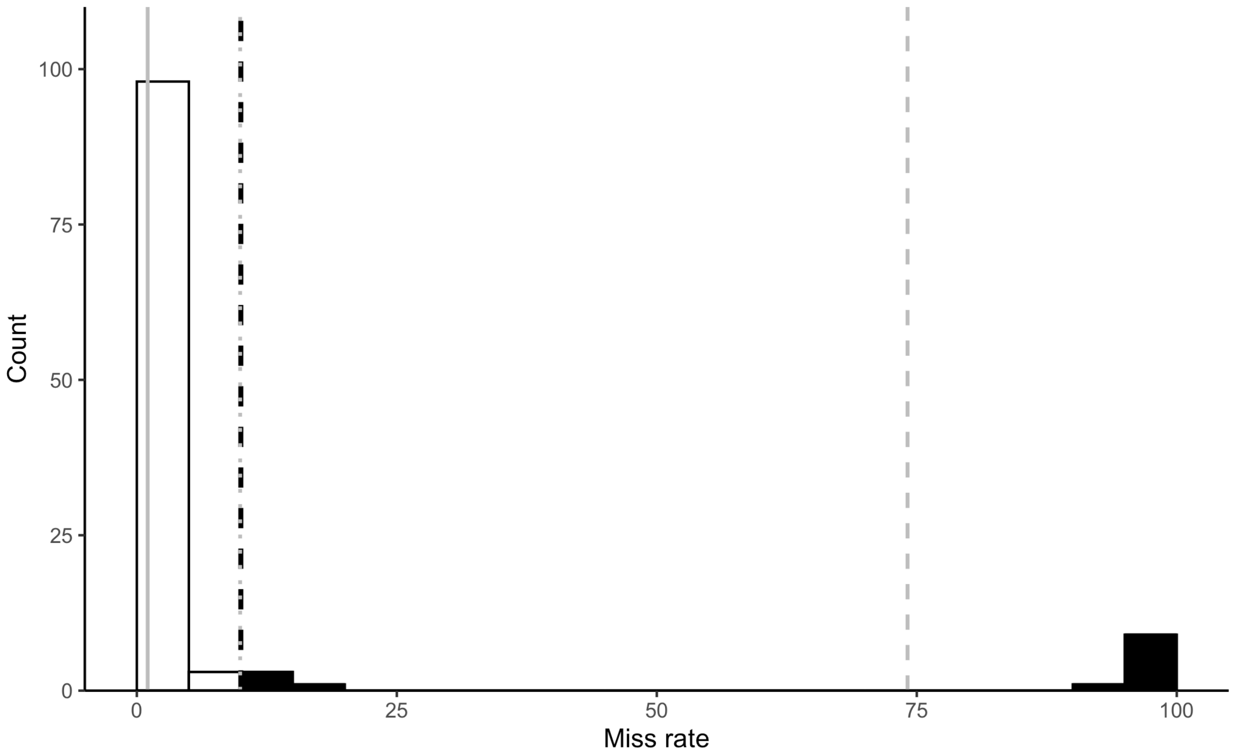

Figure S19. **Histogram of participant miss rates from Experiment 4.** The dashed black line at 10% marks the cutoff – participants with a miss rate greater than 10% were excluded. Fourteen participants had a miss rate greater than 10% (black filled bars). The dotted gray line indicates the average miss rate for all 115 participants (9.94%). The dashed gray line indicates the average miss rate for the 14 excluded participants (74.11%). The solid gray line indicates the average miss rate for the 101 included participants (1.04%). A Welch independent samples *t*-test revealed a significantly larger miss rate for the excluded participants than the 101 included participants, $t(13.01) = 6.88$, $p < .001$, $d = 5.40$.

Experiment 4 – Additional Direction Angle Results

The interaction between cue format and direction angle was not significant, $F(4,400) = 1.03$, $p = .391$, $\eta_p^2 = 0.01$, $BF_{10} = 0.003$.

The interaction between trial type, cue format, and direction angle was not significant, $F(4,400) = 0.28$, $p = .894$, $\eta_p^2 = 0.003$, $BF_{10} = 0.006$ (See Table S9 for pairwise comparisons between valid and invalid trials as a function of cue format and direction angle.) Notably, RTs on valid trials were significantly faster than RTs on invalid trials for orthogonal direction angles in all three cue formats. Moreover, the only significant difference in cue validity effects was between orthogonal and slight direction angles for word cues, $t(100) = 2.69$, $p = .008$, $d = 0.32$, $BF_{10} = 3.32$ (all other comparisons, *t*s < 2.0, *p*s > .05; see Figure S20). In general, these results indicate that cue validity effects occurred reliably for both orthogonal spatial directions and schema cues regardless of direction angle.

Table S9. *RTs as a function of cue format, direction angle, and trial type in Experiment 4.*

Cue Format	Direction Angle	*M* (*SEM*) RT for Valid Trials (ms)	*M* (*SEM*) RT for Invalid Trials (ms)	*M* (*SEM*) Cue Validity Effect (ms)	*t*	*p*	*d*	JZS Bayes factor (BF_{10})
Scene	**Orthogonal**	**392.89 (5.86)**	**401.82 (6.43)**	**8.93 (3.85)**	**2.32**	**.023**	**0.14**	**1.41**
	Sharp	395.20 (6.04)	398.82 (6.72)	3.63 (3.17)	1.14	.255	0.06	0.21
	Slight	397.00 (6.21)	398.92 (6.29)	1.91 (2.87)	0.67	.506	0.03	0.14
Schema	**Orthogonal**	**386.14 (6.42)**	**400.71 (6.61)**	**14.56 (4.14)**	**3.52**	**< .001**	**0.22**	**32.66**
	Sharp	*389.97 (5.83)*	*395.86 (6.01)*	*5.89 (2.85)*	*2.07*	*.041*	*0.10*	*0.84*
	Slight	*391.63 (5.87)*	*398.76 (6.28)*	*7.12 (3.43)*	*2.08*	*.040*	*0.12*	*0.86*
Word	**Orthogonal**	**387.25 (6.23)**	**400.17 (5.79)**	**12.92 (3.80)**	**3.40**	**< .001**	**0.21**	**22.89**
	Sharp	390.53 (6.03)	395.78 (6.29)	5.25 (3.87)	1.36	.178	0.08	0.27
	Slight	389.52 (6.07)	390.89 (5.80)	0.83 (3.78)	0.22	.826	0.01	0.11

Note: Significant differences between valid and invalid trials (cue validity effects) are bolded and marginal effects are italicized (critical $\alpha = .035$).

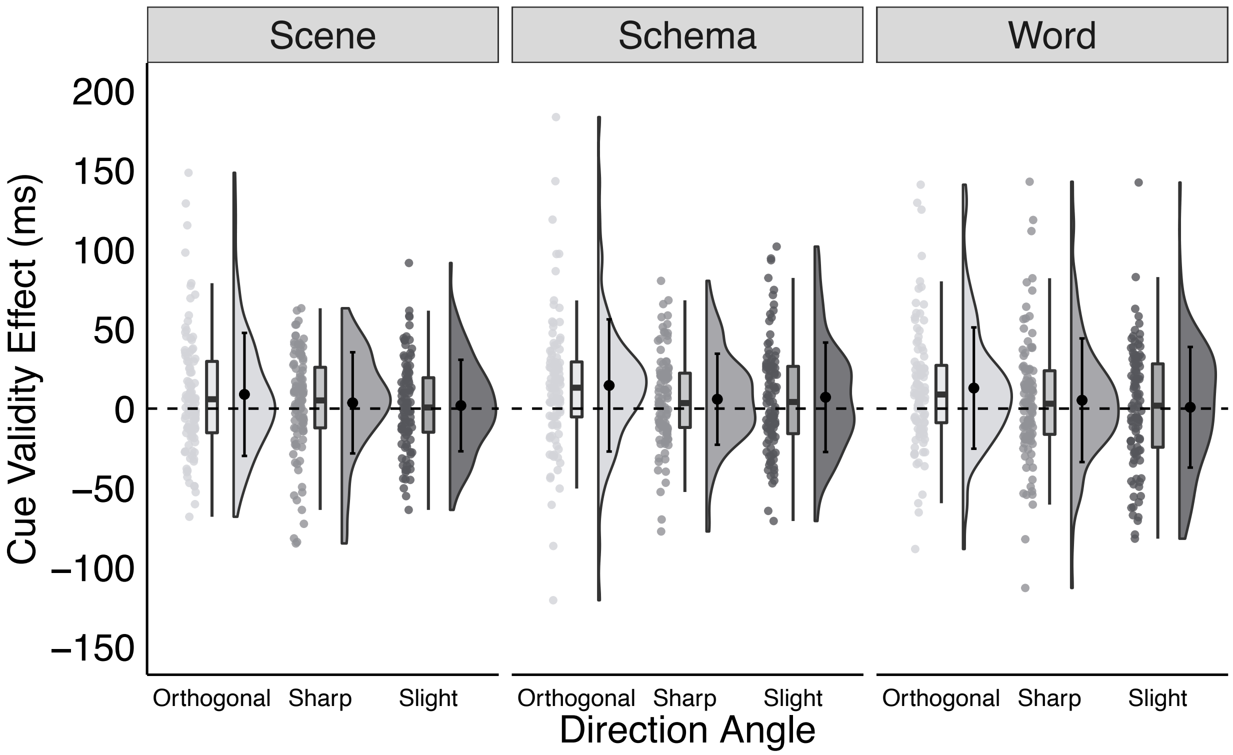

Figure S20. **No significant interaction between trial type, cue format, and cue direction in Experiment 4**, plotted as the cue validity effect as a function of cue direction for each cue format.

Inconsistent with Experiments 1-3, and not in support of our third pre-registered prediction, the interaction between trial type and cue format was not significant, $F(2,200) = 1.28$, $p = .280$, $\eta_p^2 = 0.01$, $BF_{10} = 0.04$. Planned comparisons revealed that RTs were significantly faster on valid ($M_{scene} = 395.03$ ms, $SEM_{scene} = 3.48$ ms; $M_{schema} = 389.25$ ms, $SEM_{schema} = 3.48$ ms; $M_{word} = 389.10$ ms, $SEM_{word} = 3.52$ ms) than invalid ($M_{scene} = 399.85$ ms, $SEM_{scene} = 3.73$ ms; $M_{schema} = 398.44$ ms, $SEM_{schema} = 3.63$ ms; $M_{word} = 395.44$ ms, $SEM_{word} = 3.44$ ms) trials for all three cue formats, *t*s > 2.3, *p*s < .02, *d*s > 0.08, BF_{10}s > 1.40. That is, significant cue validity effects emerged for all three cue formats. All three cue validity effects ($M_{scene} = 4.82$ ms, $SEM_{scene} = 1.92$ ms; $M_{schema} = 9.19$ ms, $SEM_{schema} = 2.03$ ms; $M_{word} = 6.34$ ms, $SEM_{word} = 2.22$ ms) were statistically equivalent across cue format, all *t*s < 1.6, all *p*s > .13 (see Figure S21),

indicating that space-based attention was preferentially allocated with equal efficiency for all cue formats.

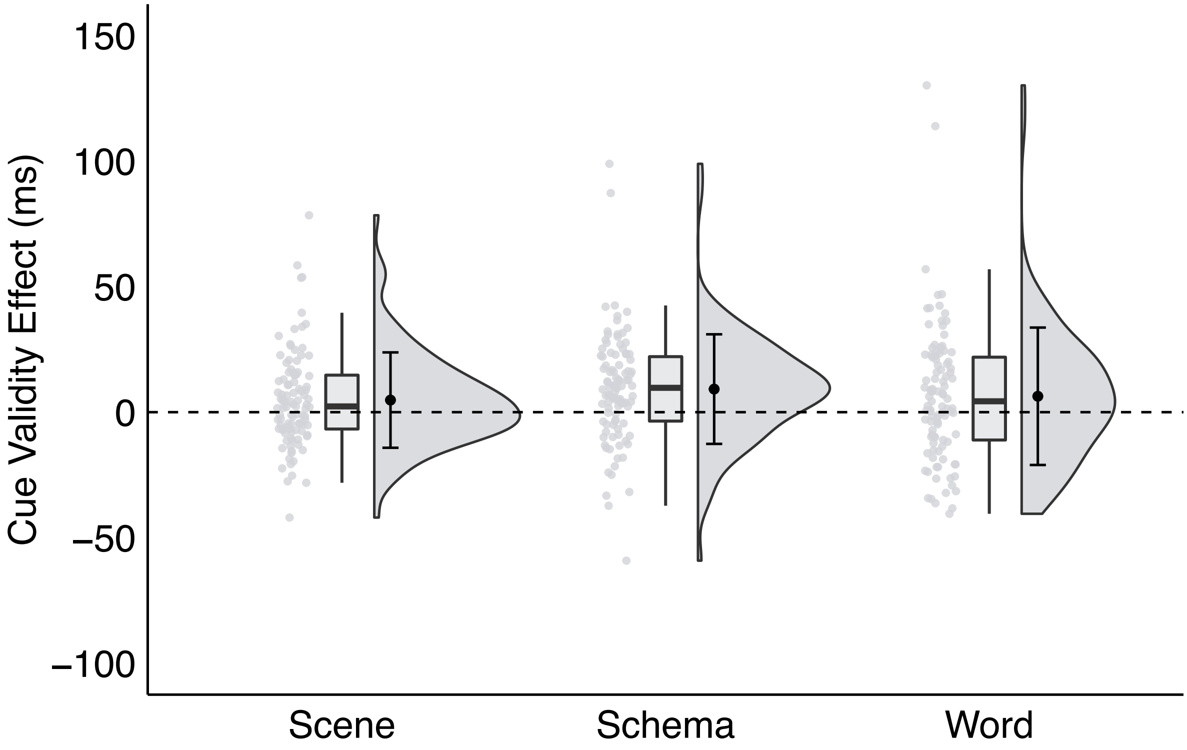

Figure S21. **No significant interaction between trial type and cue format in Experiment 4**, plotted as the cue validity effect as a function of cue format.

Experiment 4 – Exploratory Results

To explore differences in how attention was allocated with cues that provided spatial information of the upcoming target (i.e., left, right, sharp left, sharp right, slight left, and slight right spatial directions) compared to cues that did not provide any spatial information of the upcoming target (i.e., ahead spatial direction; neutral trials), RTs were submitted to a 2 (target location: left, right) x 7 (cue direction: ahead, left, right, sharp left, sharp right, slight left, slight right) x 3 (cue format: scene, schema, word) within-subjects repeated measures ANOVA. There was no main effect of cue direction or interaction between cue direction and cue type, Fs < 0.9, ps > .60. There was a significant main effect of target location, $F(1,100) = 4.90$, $p = .029$, $\eta_p^2 = 0.05$, $BF_{10} = 54.31$, such that RTs were significantly faster when the target appeared on the right ($M = 392.67$ ms, $SEM = 1.41$ ms) than on the left ($M = 396.67$ ms, $SEM = 1.46$ ms). There was also a main effect of cue format ($M_{scene} = 397.26$ ms, $SEM_{scene} = 1.80$ ms; $M_{schema} = 393.98$ ms, $SEM_{schema} = 1.75$ ms; $M_{word} = 392.76$ ms, $SEM_{word} = 1.73$ ms), $F(2,200) = 7.50$, $p < .001$, $\eta_p^2 = 0.07$, $BF_{10} = 2.11$. The interaction between target location and cue format was also significant, $F(2,200) = 4.96$, $p = .008$, $\eta_p^2 = 0.05$, $BF_{10} = 0.28$.

Consistent with Experiments 1-3, the interaction between target location and cue direction was significant, $F(6,600) = 7.80$, $p < .001$, $\eta_p^2 = 0.07$, $BF_{10} = 684{,}605.40$. This interaction was driven by differences in RTs on trials with left, right, sharp right, and slight right cues as a function of target location (see Figure S22). For trials with a left cue, RTs were significantly faster when the target appeared on the left than the right. In contrast, for trials with right, sharp right, or slight right cues, RTs were significantly faster when the target appeared on the right than the left. That is, space-based attention was preferentially allocated to the left following a left cue, resulting in faster detection of the target on the left than the right; the

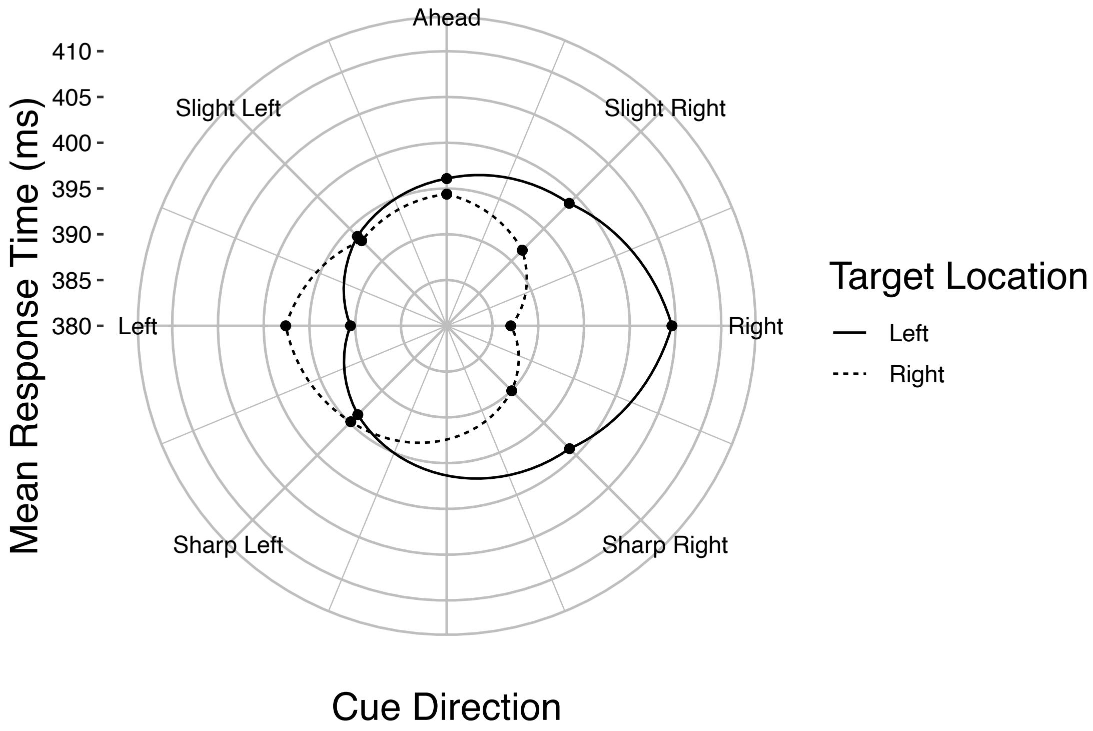

Figure S22. **Exploratory results from Experiment 4.**

reverse was true for targets on the right following a right cue, in general, regardless of the direction angle. On trials with sharp left, slight left, and, critically, ahead cues, RTs were statistically equivalent when the target appeared on the left or the right. As expected, ahead cues, as well as sharp left and slight left cues, did not provide an attentional benefit for detecting the target (see Table S10).

Unlike in Experiments 1-3, however, the interaction between target location, cue direction, and cue format was not significant, $F(12,1200) = 0.87$, $p = .578$, $\eta_p^2 = 0.01$, $BF_{10} = 0.0002$ (see Figure S23 and Table S11 in the supplementary materials).

Table S10. *RTs as a function of cue direction and target location in Experiment 4.*

Cue Direction	*M* (*SEM*) RT for Left Target Trials (ms)	*M* (*SEM*) RT for Right Target Trials (ms)	*t*	*p*	*d*	JZS Bayes factor (BF_{10})
Ahead	396.08 (3.63)	394.38 (3.64)	0.72	.470	0.03	0.08
**Left**	**390.52 (3.64)**	**397.59 (3.73)**	**2.47**	**.014**	**0.11**	**1.27**
**Right**	**404.61 (4.36)**	**387.00 (3.65)**	**5.36**	**< .001**	**0.25**	**52,542.20**
Sharp Left	393.73 (3.63)	394.82 (4.10)	0.41	.679	0.02	0.07
**Sharp Right**	**398.93 (4.02)**	**390.04 (3.48)**	**3.01**	**.003**	**0.13**	**5.34**
Slight Left	393.81 (3.63)	393.15 (3.86)	0.24	.811	0.01	0.07
**Slight Right**	**398.93 (4.02)**	**391.68 (3.60)**	**2.56**	**.011**	**0.11**	**1.59**

Note: Significant differences between left and right targets are bolded (critical α = .035).

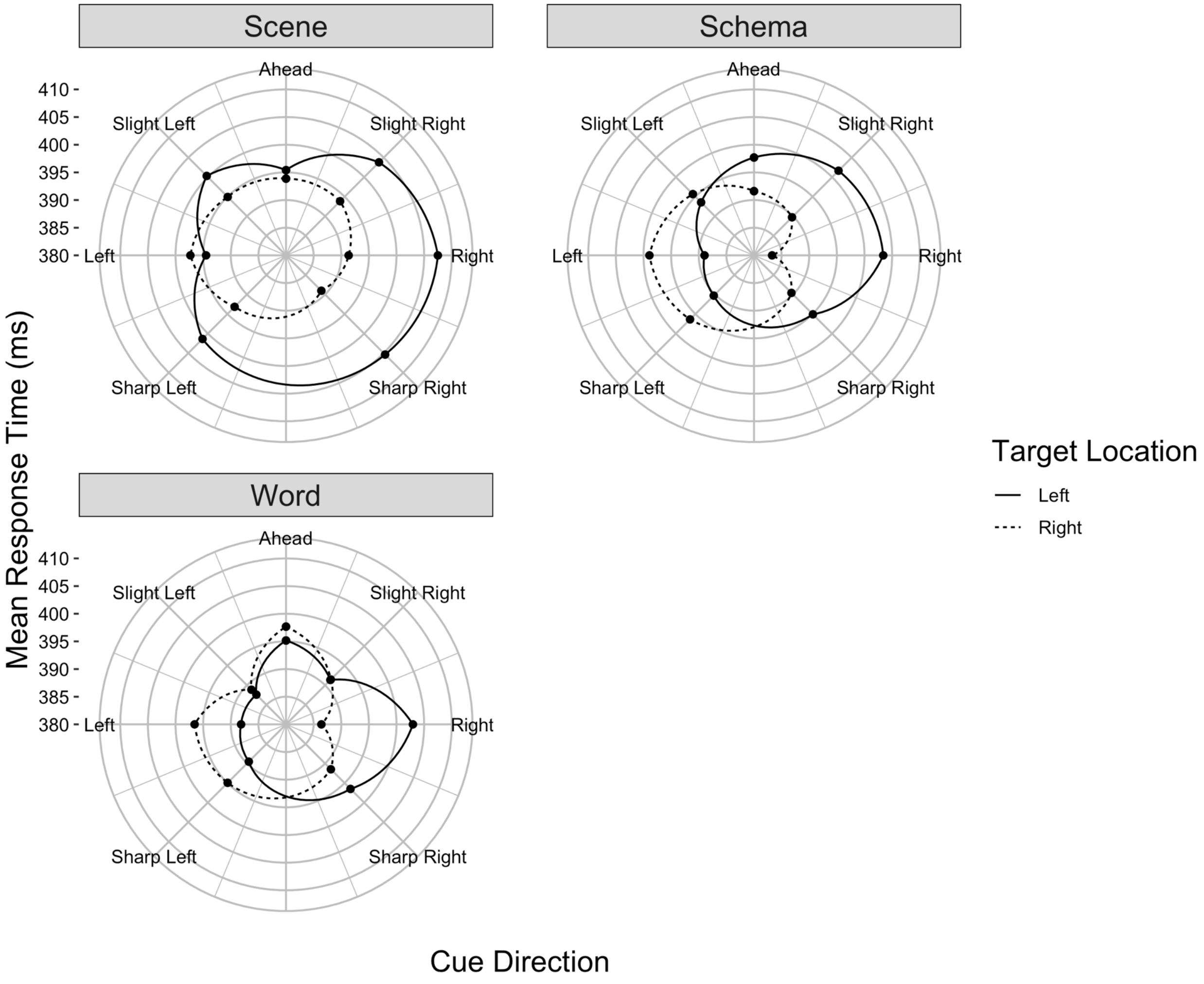

Figure S23. **Exploratory results from Experiment 4.**

Table S11. *RTs as a function of cue format, cue direction, and target location in Experiment 4.*

Cue Format	Cue Direction	*M* (*SEM*) RT for Left Target Trials (ms)	*M* (*SEM*) RT for Right Target Trials (ms)	*t*	*p*	*d*	JZS Bayes factor (BF_{10})
	Ahead	395.40 (6.06)	393.87 (6.19)	0.39	.700	0.02	0.12
	Left	394.47 (6.07)	397.31 (6.42)	0.56	.580	0.05	0.13
	**Right**	**407.48 (8.13)**	**391.33 (5.96)**	**2.76**	**.007**	**0.21**	**3.98**
Scene	Sharp Left	401.37 (6.56)	393.15 (7.70)	1.72	.088	0.11	0.46
	**Sharp Right**	**405.36 (7.46)**	**389.05 (5.95)**	**3.13**	**.002**	**0.23**	**10.34**
	Slight Left	400.30 (6.61)	394.93 (6.92)	1.17	.244	0.08	0.21
	Slight Right	*403.79 (7.31)*	*393.84 (6.34)*	*1.96*	*.053*	*0.14*	*0.69*
	Ahead	397.69 (6.26)	391.61 (6.27)	1.38	.170	0.10	0.28
	Left	*388.95 (6.69)*	*398.92 (6.87)*	*2.12*	*.036*	*0.15*	*0.94*
	**Right**	**403.39 (7.45)**	**383.28 (6.43)**	**3.44**	**< .001**	**0.28**	**25.84**
Schema	Sharp Left	390.29 (6.10)	396.36 (6.86)	1.48	.142	0.09	0.32
	Sharp Right	395.08 (6.38)	389.60 (5.92)	1.17	.244	0.09	0.21
	Slight Left	393.54 (6.07)	395.66 (6.88)	0.44	.658	0.03	0.12
	**Slight Right**	**401.64 (7.07)**	**389.73 (6.09)**	**2.35**	**.021**	**0.18**	**1.51**
	Ahead	395.16 (6.60)	397.67 (6.50)	0.66	.514	0.04	0.14
	Left	388.13 (6.18)	396.55 (6.15)	1.65	.102	0.14	0.41
	**Right**	**402.97 (7.13)**	**386.40 (6.61)**	**3.06**	**.003**	**0.24**	**8.61**
Word	Sharp Left	389.53 (6.15)	394.94 (6.75)	1.16	.249	0.08	0.21
	Sharp Right	396.50 (7.16)	391.48 (6.26)	0.92	.362	0.07	0.17
	Slight Left	387.59 (6.15)	388.85 (6.26)	0.25	.806	0.02	0.11
	Slight Right	391.35 (6.47)	391.46 (6.30)	0.02	.981	0.002	0.11

Note: Significant differences between left and right targets are bolded, and marginal differences are italicized (critical $\alpha = .035$).

Combined Analysis

To strengthen confidence in the findings for our three pre-registered predictions, we combined the data from Experiments 1-4 into a common dataset based on shared conditions across all experiments – orthogonal (i.e., left and right) spatial directions and 1200 ms cue duration. RTs were collapsed across target location and cue direction and submitted to a 2 (trial type: valid, invalid) x 3 (cue format: scene, schema, word) within-subjects repeated measures ANOVA.

Pre-registered Prediction 1: Faster performance on valid than invalid trials

We found a main effect of trial type, $F(1,402) = 84.92$, $p < .001$, $\eta_p^2 = 0.17$, $BF_{10} = 8.32^{20}$, such that RTs were significantly faster on valid ($M = 388.66$ ms, $SEM = 1.57$ ms) than invalid ($M = 399.78$ ms, $SEM = 1.78$ ms) trials. Across all experiments, space-based attention was preferentially allocated to targets in validly cued over invalidly cued locations, reflecting a robust cue validity effect.

Pre-registered Prediction 2: Faster overall performance with schema cues than word and scene cues

We found no main effect of cue format ($M_{scene} = 393.86$ ms, $SEM_{scene} = 1.99$ ms; $M_{schema} = 394.14$ ms, $SEM_{schema} = 2.10$ ms; $M_{word} = 394.67$ ms, $SEM_{word} = 2.09$ ms), $F(2,804) = 0.21$, $p = .810$, $\eta_p^2 = 0.0005$, $BF_{10} = 0.006$ (all pairwise comparisons, *t*s < 0.7, *p*s > .53). In general, target detection speed was not influenced by cue format.

Pre-registered Prediction 3: Modulation of cue validity effects by cue format

The interaction between trial type and cue format was significant, $F(2,804) = 16.32$, $p < .001$, $\eta_p^2 = 0.04$, $BF_{10} = 21{,}925.25$. This interaction was driven by a difference in RTs on trials with either a schema or word cue as a function of trial type. Specifically, for schema cues, RTs

were significantly faster on valid (*M* = 385.59 ms, *SEM* = 2.79 ms) than invalid (*M* = 402.69 ms, *SEM* = 3.09 ms) trials, $t(402) = 8.88$, $p < .001$, $d = 0.29$, $BF_{10} = 1.93^{14}$. Similarly, for word cues, RTs were significantly faster on valid (*M* = 388.09 ms, *SEM* = 2.71 ms) than invalid (*M* = 401.26 ms, *SEM* = 3.16 ms) trials, $t(402) = 6.95$, $p < .001$, $d = 0.22$, JZS BF_{10} = 373,278,528. However, with scene cues, RTs were statistically equivalent on valid (*M* = 392.31 ms, *SEM* = 2.64 ms) and invalid (*M* = 395.40 ms, *SEM* = 2.97 ms) trials, $t(402) = 1.66$, $p = .098$, $d = 0.05$, $BF_{10} = 0.22$. That is, significant cue validity effects were observed for schema and word cues, but not scene cues.

To follow up, we conducted planned pairwise comparisons between the three cue validity effects (see Figure S24). Specifically, the schema cue validity effect (*M* = 17.10 ms, *SEM* = 1.93

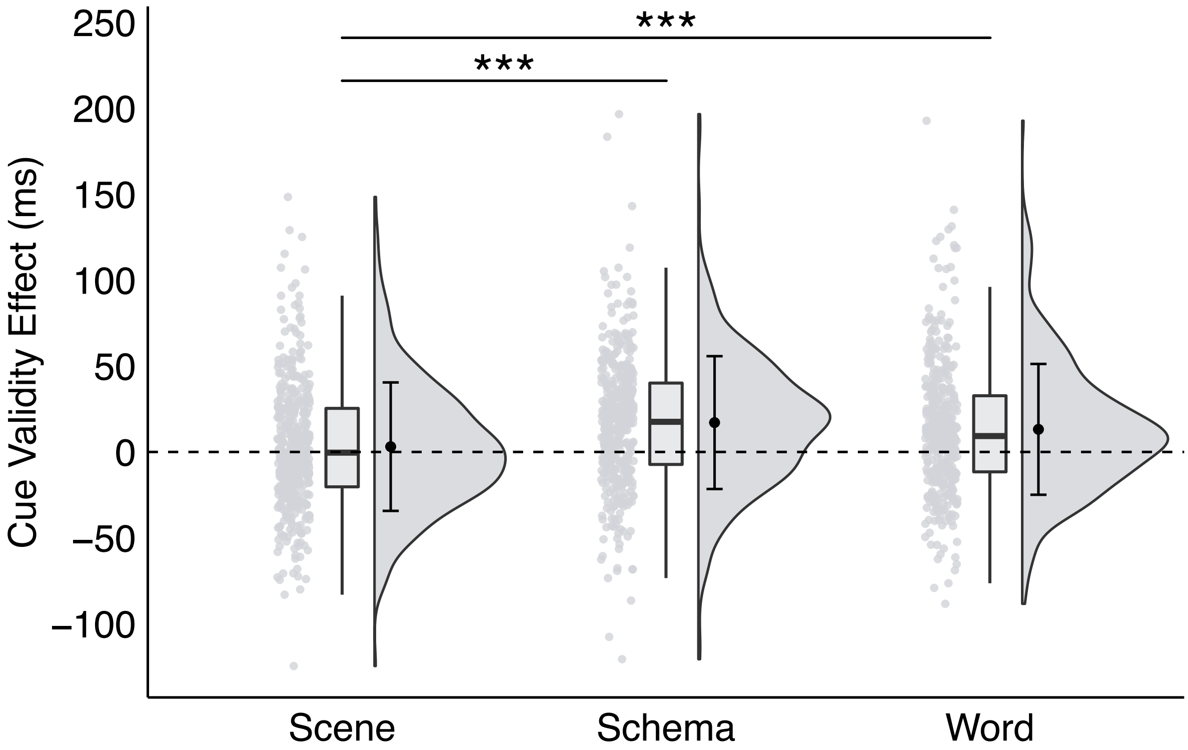

***Figure S24.* A significant interaction between trial type and cue format when data are combined across Experiments 1-4**, plotted as the cue validity effect as a function of cue format. *Note*: * $p < .035$, ** $p < .01$, *** $p < .001$; critical $\alpha = .035$.

ms) and the word cue validity effect (M = 13.17 ms, SEM = 1.90 ms) were significantly larger than the scene cue validity effect (M = 3.09 ms, SEM = 1.86 ms), $t(402) = 5.41$, $p < .001$, d = 0.37, BF_{10} = 68,450.17 and $t(402) = 3.95$, $p < .001$, $d = 0.27$, BF_{10} = 110.47, respectively. The schema cue validity effect was not significantly different from the word cue validity effect, $t(402) = 1.61$, $p = .108$, $d = 0.10$, $BF_{10} = 0.20$. In general, the preferential allocation of space-based attention was greater (and more efficient) for schema and word cues than scene cues.
